# Comparison of chest compression quality in walking versus straddling cardiopulmonary resuscitation during stretcher transportation: A prospective randomised crossover study using manikins

**DOI:** 10.1371/journal.pone.0216739

**Published:** 2019-05-21

**Authors:** Mikako Shinchi, Masanao Kobayashi, Kaori Soma, Akifumi Maeda

**Affiliations:** 1 Department of Nursing, Hirakata City Hospital, Hirakata, Osaka, Japan; 2 Department of Emergency Medicine, Hirakata City Hospital, Hirakata, Osaka, Japan; Weill Cornell Medical College, UNITED STATES

## Abstract

The optimal strategy to ensure chest compression quality for patients being transported on a stretcher has not been established yet. We hypothesised that straddling cardiopulmonary resuscitation may improve chest compression quality in patients being transported on stretchers. We conducted a prospective randomised crossover study using manikins to investigate whether straddling cardiopulmonary resuscitation improves chest compression quality (depth, recoil, rate, correct hand position) performed on patients during stretcher transportation compared to walking cardiopulmonary resuscitation. Walking and straddling cardiopulmonary resuscitation were performed for 2 minutes each. The mean chest compression depth (mm) for 2 minutes was significantly greater in the straddling cardiopulmonary resuscitation group than in the walking cardiopulmonary resuscitation group (median, 51.3 [interquartile range, 46.7–55.5] versus 40.9 [34.6–50.1], P = 0.003). An adequate depth of chest compressions could not be achieved when walking cardiopulmonary resuscitation was performed by female participants, but the depth of chest compressions was within the acceptable range when female participants performed straddling cardiopulmonary resuscitation. On the other hand, the degree of deterioration was relatively small in male participants, even when they performed walking cardiopulmonary resuscitation. In patients with cardiac arrest being transported on a stretcher, straddling cardiopulmonary resuscitation improved the depth of chest compressions compared to walking cardiopulmonary resuscitation. Female rescuers, in particular, may consider using straddling cardiopulmonary resuscitation.

## Introduction

### Background

High-quality chest compressions are essential, even when patients with cardiac arrest have to be transported. In-hospital cardiac arrest can occur anywhere in the hospital, including the ward and places such as hallways and cafeterias. There are few procedures that can be performed in situations where a resuscitation device is not readily available, and patients should be transported to the emergency department or a treatment room where advanced procedures can be performed as soon as possible. Additionally, cardiopulmonary resuscitation (CPR) may need to be performed while patients are being transported to the angiography room for extracorporeal CPR (venoarterial extracorporeal membrane oxygenation).

Constant high-quality chest compressions are undoubtedly required during transportation because the prognosis of the patient depends on the quality of CPR. The patients are transported on a stretcher in most cases. However, it is reportedly difficult to ensure CPR quality is maintained while walking (hereafter “walking CPR”) during stretcher transportation. Even with an excellent resuscitation team, it is difficult to ensure the quality of chest compressions during stretcher transportation.

Kim et al. [1] reported that chest compressions performed on a moving stretcher were significantly shallower than those performed on the floor (mean compression depth was 39 [standard deviation (SD), 9] mm versus 28 [SD, 9] mm, respectively; the mean difference was 11 mm, *P* < 0.001). To overcome this problem, CPR may be performed while straddling the patient (hereafter “straddling CPR”). Handley et al. (2004) reported that straddling CPR, which can be implemented even in confined spaces, is as effective as normal CPR [2]. The report by Lei et al. [3] is believed to be the first on straddling CPR performed with the aim of ensuring chest compression quality during transportation. In this study, chest compressions similar to those performed on the floor were performed safely by straddling CPR on a moving stretcher (mean compression depth was 43.4 [SD, 3.6] mm versus 43.7 [SD, 3.3] mm, respectively; *P* = 0.78). The best strategy to ensure optimal chest compression quality for patients being transported on a stretcher has not yet been established; moreover, there are no studies comparing straddling CPR and walking CPR, although these strategies are required in all hospitals. Therefore, determining whether straddling CPR is superior to walking CPR is extremely important. We think that straddling CPR should become more widely known and practiced. Straddling CPR is not difficult to perform; it can be performed immediately in case of an emergency. If the superiority of straddling CPR is proved, we believe that it should be promoted as a useful strategy to maintain the quality of chest compressions during stretcher transportation.

### Purpose

We used manikins to examine whether chest compression quality (depth, recoil, rate, and correct hand position) improved in straddling versus walking CPR for patients with cardiac arrest being transported on a stretcher.

## Materials and methods

### Participants and setting

We recruited doctors and nurses from our institution (335-bed hospital visited by patients with acute stage disease) as volunteer participants. All participants had participated in and were certified in a 3-hour basic life support course (hospital certified) or a 7-hour immediate cardiac life support course (certified by the Japanese Association for Acute Medicine) within the past 3 years.

### Study design

Between 9 January and 23 January 2018, we conducted a prospective randomised crossover study using manikins. The participants were asked to perform walking CPR and straddling CPR. The main outcome was the quality of chest compressions. The study complied with the Declaration of Helsinki and was approved by the Research Ethics Committee of Hirakata City Hospital. The participants were given an oral explanation and were obliged to read the written study protocol. The participants who signed a consent form after reaching an agreement with the researchers were included in the study.

### Pilot study

As a pilot study, we had 10 participants perform straddling CPR and walking CPR and compared the results, following the same protocol as the present study, which will be discussed later. The mean difference in compression depth during 2 minutes was 5.8 mm. The Prospective Sample Size and Power of JMP Pro 13 (SAS Institute Inc., Cary, North Carolina, USA) was used to calculate that the required sample size was 41 cases (α = 0.05, power = 0.8, m = 1, δ = 5.8). Based on these results, we recruited 22 participants.

### Study protocol

**Fig 1:**
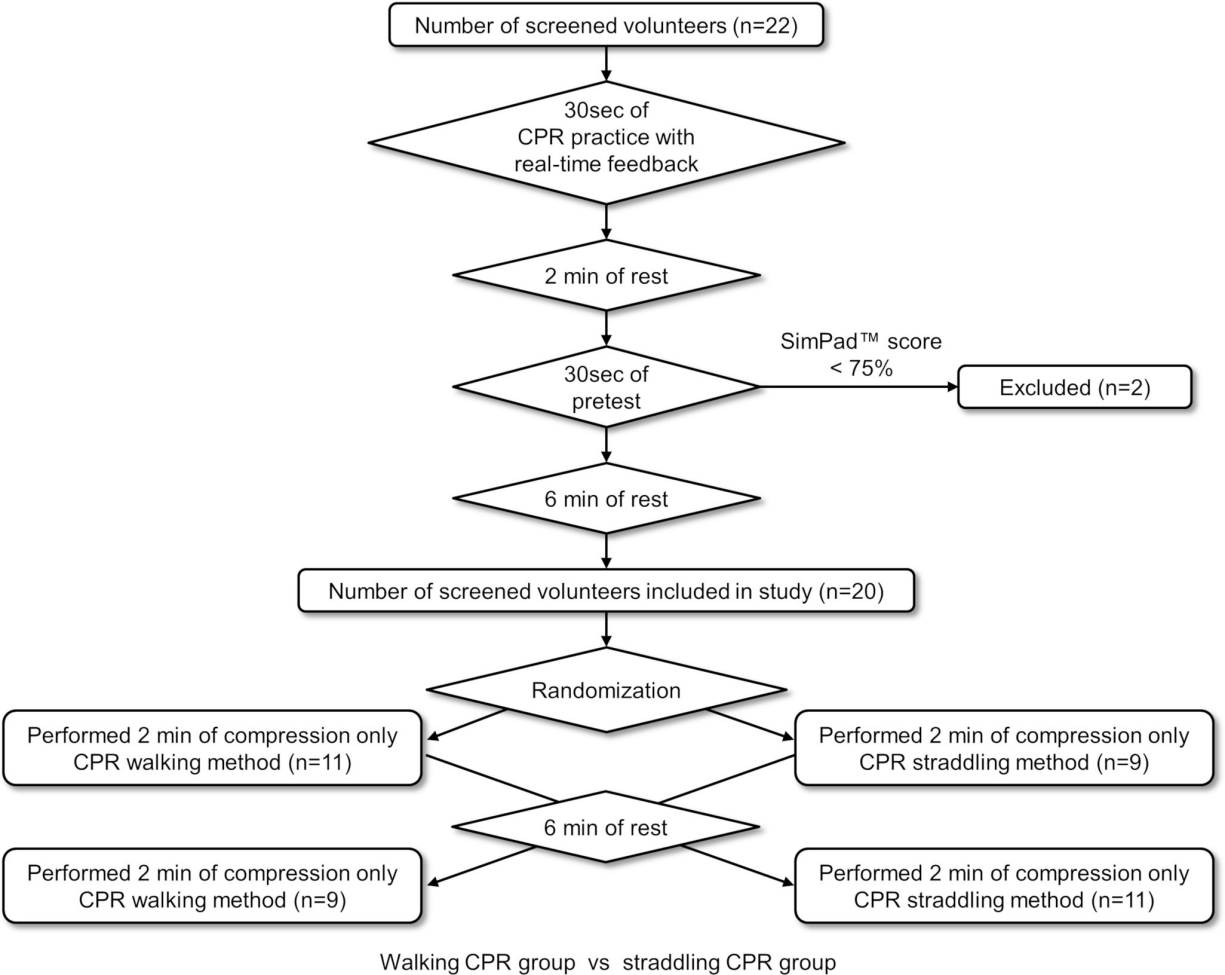
Study protocol and flow diagram. Flow diagram illustrating inclusion and exclusion criteria of participants in the randomised crossover study.

The study protocol and flow diagram are shown in Fig 1. A video clip demonstrating straddling and walking CPR can be viewed at: https://goo.gl/LNAAYN. A simulator (Resusci-Anne Simulator SimPad version, Laerdal Medical, Stavanger, Norway; weight, 36 kg) was placed on a solid steel stretcher without springs (model number NV-STR, As One Co., Osaka, Japan; width, 58 cm; capacity, 150 kg). This stretcher does not bounce with chest compressions. Moreover, the stretcher mat is only 1.5 cm thick, and it does not sink with chest compressions. These features are considered to be important not only in research but also in clinical practice. The participants were asked to stand next to the manikin. The height of the stretcher was adjusted so that the shoulders of the participants were located right above the sternum of the manikin with the aim of maximising the efficiency of the chest compressions [4]. To review the correct depth, recoil, rate, and hand position, we let the participants practice real-time feedback CPR for 30 seconds. Next, in order to exclude the participants who could not perform chest compressions adequately, a pretest that was 30 seconds in duration was conducted after 2 minutes of rest. The 2-minute rest period and 30-second duration of pretest were used to ensure that there was no fatigue. In the pretest, compression only CPR was performed for 30 seconds. Participants whose compression score [5] on the SimPad was < 75% were excluded from the study. This is because the manufacturer defines a compression score of 75% to 100% as an “Advanced CPR Performer” (passing score). The chest compression depth was set on the SimPad at a lower limit of 45 mm and an upper limit of 60 mm. This score was only used for an exclusion criterion. Of the 22 participants who participated in the present study, two were excluded based on the pretest results.

Walking CPR and straddling CPR were performed for 2 minutes each, and the chest compression quality was examined after a rest ≥ 6 minutes [6]. We set the CPR time to 2 minutes because the European Resuscitation Council Guidelines for Resuscitation 2015 recommend that CPR providers be changed about every 2 minutes to prevent a decrease in compression quality due to CPR provider fatigue [7]. We have no data on the length of transport time for in-hospital cardiac arrest, but in our experience, transport is typically completed within 2 minutes. Normally, the rescuer will not be replaced during this time. Therefore, we decided to measure chest compressions for 2 minutes while transporting one participant. The envelope method was used to randomly determine whether walking CPR or straddling CPR would be performed first. A paper reading “straddling CPR first" or "walking CPR first" was drawn from the envelope. The height of the stretcher was set at the same height as in the pretest for both walking CPR and straddling CPR for each participant. The stretcher was transported at an average walking speed on the flat, smooth, rigid vinyl floor of the hospital. Walking CPR was performed with the participants continuously performing chest compressions while walking. The first author pushed the stretcher to adjust the speed. In straddling CPR, chest compressions and transportation were started after the participants climbed on the stretcher upon being signalled by a researcher to start. To allow the participants to recover, the second CPR technique was performed after a rest ≥ 6 minutes [6]. A metronome was set at 110 bpm to assist with setting the rate for CPR. We used a metronome because most automated external defibrillators have a metronome function. The greatest bias in this study was that the participants were not blinded to the two CPR methods. In order to minimise this bias, the researchers pushing the stretcher constantly encouraged the participants to ‘push harder’.

Data recorded on the SimPad were imported into Microsoft Excel (Microsoft Corporation, Redmond, Washington, USA) to calculate the depth, recoil (leaning depth), rate, and correct hand position of each compression. Based on a mean 225 pieces of compression data for each participant, the mean values were calculated for 2 minutes, and the following variables were used: first, the period between 0 and 30 seconds; second, the period between 30 and 60 seconds; third, the period between 60 and 90 seconds; and fourth, the period between 90 and 120 seconds.

### Statistical analysis

JMP Pro 13 was used for the statistical analysis. The Shapiro-Wilk test was used to test the normality of the distribution of each group. Because only partial normality was obtained, Wilcoxon/Kruskal-Wallis tests (rank sums) were used to compare the walking CPR group and straddling CPR group, while the Steel-Dwass test was used to compare the variables of each period. The chi-square test was used for 2 × 2 table analysis. The data are represented as medians [interquartile range: IQR].

## Results

### Characteristics of study participants

The characteristics of the study participants are shown in Table 1. The results of 20 participants (10 male and 10 female participants) were examined. All 10 female participants were nurses, and 9 of the 10 male participants were doctors. Owing to this serious bias, we could not discuss whether the difference in occupation was related to the quality of chest compressions. The characteristics of the study participants are listed in Table 1. There was a statistically significant difference in the transportation speed (m/min) between the walking CPR group and the straddling CPR group; however, while this was a statistical difference, it was considered not to be a clinical difference.

**Table 1:** Characteristics of study participants.

	Walking CPR (n = 20)	Straddling CPR (n = 20)	P-value
Age—years, median [IQR *Q*_1_-*Q*_3_]	36 [28–41.8]		-
Sex—no. [male participants : female participants]	10 : 10		1.00*
Participants—occupation [doctor : nurse]	9:11		1.00*
Height—cm, median [IQR *Q*_1_-*Q*_3_]	164 [157.3–173]		-
Weight—kg, median [IQR *Q*_1_-*Q*_3_]	64 [56.5–68.8]		-
Body mass index—kg/cm^2^, median [IQR *Q*_1_-*Q*_3_]	22.9 [20.7–24.7]		-
Stretcher’s height—cm, median [IQR *Q*_1_-*Q*_3_]	51.3 [50–53]		-
Moving speed—m/min, median [IQR *Q*_1_-*Q*_3_]	70 [70–70]	70 [70–70.4]	0.036
Time to first compression—sec, median [IQR *Q*_1_-*Q*_3_]	-	4.0 [2.7–5.6]	-

Medians [interquartile range (IQR), *Q*_1_: lower quartile (25th percentile)—*Q*_3_: upper quartile (75th percentile)]

### Main results

Table 2 shows the mean chest compression quality over a period of 2 minutes. The mean chest compression depth (mm) was significantly greater in the straddling CPR group than in the walking CPR group. When we divided the 2 minutes of CPR into four 30-second periods, the straddling CPR group showed a significantly greater mean chest compression depth (mm) than the walking CPR group in all four periods. The compression depth deteriorated with time. There were statistically significant differences between the first and third periods and between the first and fourth periods of the walking CPR group as well as between the first and fourth periods of the straddling CPR group. There was a significant difference in the mean compression rate between the walking CPR group and the straddling CPR group, and while this was a statistical difference, it was not considered to be a clinical difference. There was no significant difference in mean recoil (mm) between the walking CPR group and the straddling CPR group. The percentage of the participants with a correct hand position showed no significant intergroup difference.

**Table 2:** Quality of chest compressions.

Variable (main outcome)	Walking CPR	Straddling CPR	*P*-value
Mean compression depth, mm			
total 2 minutes	40.9 [34.6–50.1]	51.3 [46.7–55.5]	0.003
1^st^ period (0–30 seconds)	46.3 [38.6–55.5] *	55.6 [48–60.3] **	0.007
2^nd^ period (30–60 seconds)	42.1 [36.3–53.5] ^†^	52.1 [46.8–57.4] ^††^	0.009
3^rd^ period (60–90 seconds)	35.8 [30.7–46.7] ^‡^	48.8 [44.6–53.7] ^‡‡^	0.001
4^th^ period (90–120 seconds)	35.8 [32.5–45.2] ^§^	46.4 [44.8–51.7] ^§§^	0.001
Mean compression rate, /min	111.1 [110.7–113.1]	110.8 [110.3–111.1]	0.04
Mean compression recoil (leaning depth), mm	3.1 [1.1–4.8]	4.9 [0.5–7.8]	0.66
Mean correct hand position in 2 min, %	100 [100–100]	100 [100–100]	0.94

Walking CPR group vs straddling CPR group: Wilcoxon/Kruskal-Wallis tests (rank sums)

Comparison between inter-30’s section: Steel-Dwass test

Medians [interquartile range (IQR), *Q*_1_: lower quartile (25th percentile)—*Q*_3_: upper quartile (75th percentile)]

* vs †: p = 0.27, † vs ‡: p = 0.17, ‡ vs §: p = 0.94, * vs ‡: p = 0.011, † vs §: p = 0.12, * vs §: p = 0.007, ** vs ††: p = 0.49, †† vs ‡‡: p = 0.68, ‡‡ vs §§: p = 0.97, ** vs ‡‡: p = 0.07, †† vs §§: p = 0.129, ** vs §§: p = 0.016

**Fig 2:**
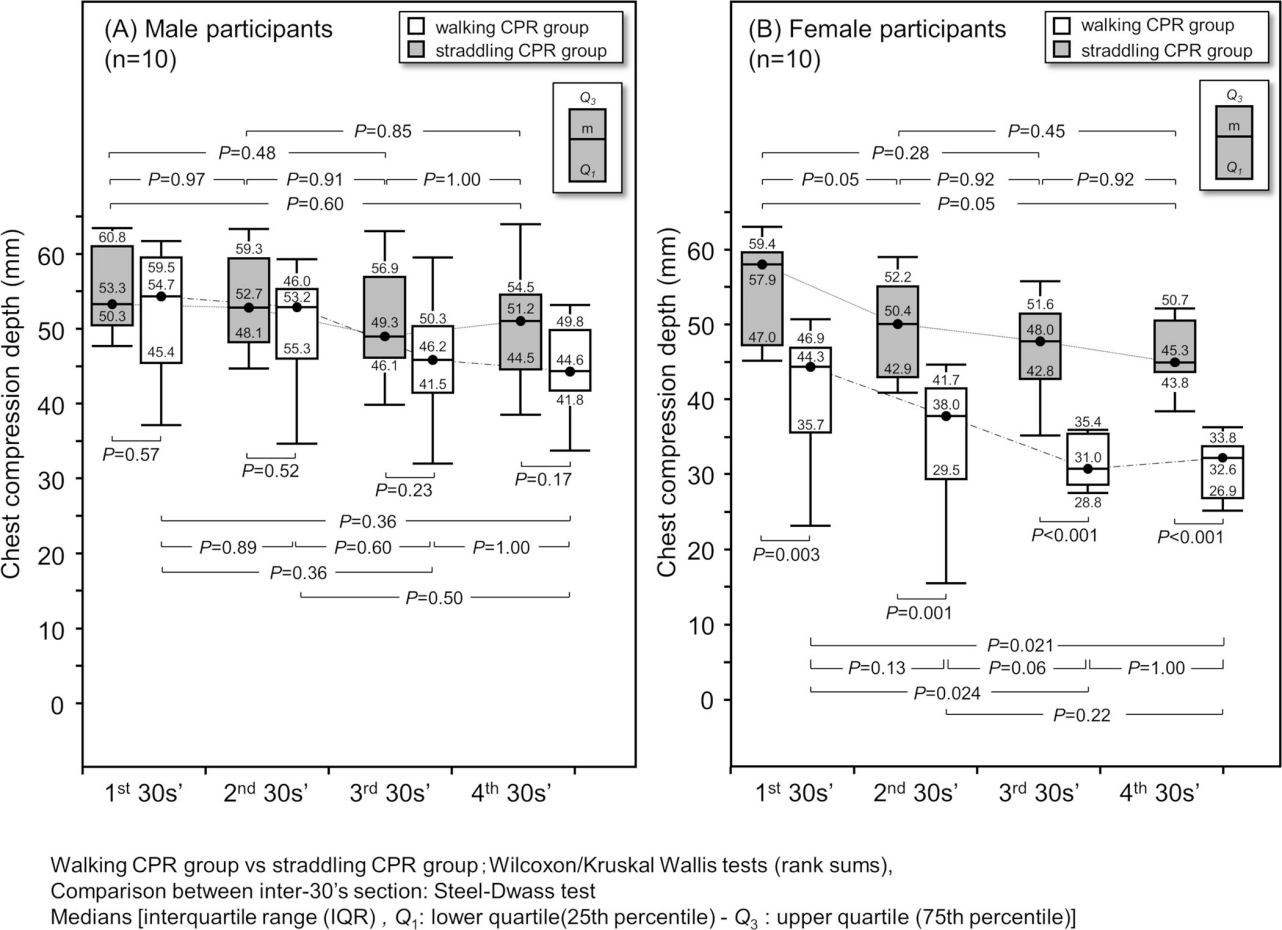
Time-dependent deterioration in mean compression depth by sex. Walking CPR group vs straddling CPR group: Wilcoxon/Kruskal-Wallis tests (rank sums); Comparison between inter-30’s section: Steel-Dwass test Medians [interquartile range (IQR), *Q*_1_: lower quartile (25th percentile)—*Q*_3_: upper quartile (75th percentile)].

We further examined the groups by sex (Fig 2) and found that there was no significant deterioration in compression depth over time in male participants, and there was no significant difference in compression depth between the straddling CPR group and the walking CPR group during any of the periods. In female participants, the straddling CPR group showed a significantly greater compression depth than the walking CPR group in all periods. Furthermore, regarding the time-dependent change for female participants in the walking CPR group, there was a significant deterioration between the first and third periods and a significant deterioration between the first and fourth periods. Meanwhile, there was no significant difference in the compression depth for female participants in the straddling CPR group although the compression depth tended to deteriorate with time.

## Discussion

### Comparison of chest compression depth

We considered the reasons why chest compressions were not performed adequately in walking CPR. To effectively perform chest compressions, the rescuer first kneels or stands by the victim. It is, then, necessary to stretch both elbows straight and to assume a posture where the rescuer’s shoulders are directly above the part to be compressed, so that weight is applied vertically on the sternum. Chest compressions are performed while maintaining this basic posture. However, in walking CPR, it is difficult for the rescuer to maintain this posture because it is necessary for the rescuer to walk while performing chest compressions. Consequently, weight cannot be applied perpendicular to the sternum; therefore, pressure may be applied using arm strength to maintain the depth of chest compressions although there is no evidence to prove this. However, Yasuda et al. [8] examined the muscles used for chest compressions in a moving ambulance using electromyography and reported that there was significantly higher activity of the triceps brachii, biceps femoris, and pectoralis major muscles; therefore, in CPR performed on an unstable floor, the effectiveness of chest compressions is dependent on the upper limb muscles. These findings suggest that walking CPR involves more muscle activity than straddling CPR and may lead to fatigue afterwards. This is the reason that the chest compression depth was significantly lower in the walking CPR group than in the straddling CPR group.

There are studies that associate chest compressions with sex. Although some studies show no difference [9–11], most studies have shown a decrease in chest compression depth when CPR is performed by female participants [12–25]. Under certain conditions, sex differences become more marked. We believe that these differences depend on the study design. We examined the sex differences in the present study and found that compression depth significantly deteriorated in female participants during walking CPR. In our study, walking CPR performed by female participants had significant attenuation in compression depth, but there were no sex differences in 30-second chest compressions performed on stationary stretchers. From this, it is considered that sex differences are difficult to detect under conditions wherein chest compressions are easy to perform. It has been reported that although there are no differences in the physiological specificity of muscles between males and females, the absolute muscle strength of females is approximately 55–70% of that of men in average Japanese adults, and women have less muscle mass in the upper torso in particular [26]. Therefore, it is believed that the sex difference in compression depth was caused by the difference in strength due to the difference in muscle mass. We assume that it is easier for male participants than it is for female participants to compensate for the disadvantages posed by walking CPR.

In addition, we set the lower limit of the adequate compression depth to 4 cm and evaluated the percentage of participants achieving that depth to examine sex differences. We set 4 cm as the lower limit because it was approved for Asians in the 2015 Resuscitation Council of Asia (RCA) guidelines [27], and Stiell et al. [28] defined the “sweet spot” as 4.03–5.53 cm. As a result, it became clearer that female rescuers were unable to achieve even 4 cm of compression depth during walking CPR. The percentage of participants achieving adequate compression depth during the last minute was as low as 1.8%, making the difference between male participants and female participants even more pronounced (Table 3). On the other hand, in straddling CPR performed by female participants, there was no time-dependent deterioration in the percentage of participants achieving adequate compression depth, and they were able to meet the minimum requirement of 4 cm established in the 2015 RCA guidelines [27]. This suggests that straddling CPR allows female rescuers to maintain chest compressions at necessary levels. On the contrary, although chest compression depth tended to deteriorate, male rescuers were able to maintain chest compressions for 2 minutes in both walking CPR and straddling CPR without a significant difference. However, straddling CPR may also be considered for male rescuers for cases in which prolonged CPR is required during transportation since compression depth tended to show a more marked deterioration in walking CPR.

**Table 3:** Time-dependent deterioration in percentage of participants achieving adequate compression depth by sex.

Sex	Time period	% Adequate compression depth		P-value
		Walking CPR	Straddling CPR	
Male	total 2 minutes	93.9 [76.0–99.3]	100 [99.4–100]	0.006
	1^st^ period (0–30 sec.)	100 [82.5–100] *	100 [99.6–100] **	0.42
	2^nd^ period (30–60 sec.)	100 [88.6–100] ^†^	100 [100–100] ^††^	0.17
	3^rd^ period (60–90 sec.)	88.2 [64.6–100] ^‡^	100 [100–100] ^‡‡^	0.006
	4^th^ period (90–120 sec.)	88.3 [72.4–97.5] ^§^	100 [99.6–100] ^§§^	0.002
Female	total 2 minutes	30.8 [8.8–43.5]	99.3 [92.4–100]	<0.001
	1^st^ period (0–30 sec.)	82.8 [25–89.9] *	100 [98.3–100] **	<0.001
	2^nd^ period (30–60 sec.)	32.4 [1.4–64.3] ^†^	100 [95.5–100] ^††^	<0.001
	3^rd^ period (60–90 sec.)	1.8 [0–15.7] ^‡^	100 [83.5–100] ^‡‡^	<0.001
	4^th^ period (90–120 sec.)	1.8 [0–6.5] ^§^	100 [87.6–100] ^§§^	<0.001

% adequate compression depth: no. of adequate compressions depth/no. of total compressions

The definition of the adequate depth is ≥40 mm, according to the 2015 Resuscitation Council of Asia guidelines.

Walking CPR group vs straddling CPR group: Wilcoxon/Kruskal-Wallis tests (rank sums)

Comparison between inter-30’s section: Steel-Dwass test

Medians [interquartile range (IQR), *Q*_1_: lower quartile (25th percentile)—*Q*_3_: upper quartile (75th percentile)]

Men: * vs †: p = 1.00, † vs ‡: p = 0.49, ‡ vs §: p = 0.10, * vs ‡: p = 0.65, † vs §: p = 0.32, * vs §: p = 0.48, ** vs ††: p = 0.97, †† vs ‡‡: p = 1.00, ‡‡ vs §§: p = 1.00, ** vs ‡‡: p = 0.095, †† vs §§: p = 1.00, ** vs §§: p = 0.92

Women: * vs †: p = .032, † vs ‡: p = 0.35, ‡ vs §: p = 0.98, * vs ‡: p = 0.007, † vs §: p = 0.18, * vs §: p = 0.008, ** vs ††: p = 0.96, †† vs ‡‡: p = 1.00, ‡‡ vs §§: p = 1.00, ** vs ‡‡: p = 0.100, †† vs §§: p = 1.00, ** vs §§: p = 0.93

### Feasibility and danger

Possible risks of straddling CPR include falls and stretcher damage. Kim et al. [1] suggested the possibility that straddling CPR during stretcher transportation may be effective, but they did not examine its safety. Meanwhile, no hazardous events occurred during the present study or in that conducted by Lei et al. [3] On the other hand, walking CPR may involve possible risks such as stumbling or having one’s foot stepped on, but no hazardous events occurred in the present study or in that conducted by Kim et al. [1] We believe that more cases must be collected to discuss the safety of straddling CPR and walking CPR, as the number of current cases is limited.

There is also currently no evidence of patient risk. Since the area where the maximum force is generated during chest compressions is the hypothenar eminence [29], Lei et al. pointed out that damage to the sternocostal junction may occur during straddling CPR [3]. There is also no general evidence regarding this issue; thus, there is a need for further research.

Performing straddling CPR may delay transportation. However, we believe it would not create clinical issues since it only took a median of 4.0 [IQR, 2.7–5.6] seconds from the point at which the rescuer started to straddle to the point where he/she started to perform compressions in the present study.

Regarding feasibility, the width of the stretcher used in the present study was 58 cm, and the width of the simulator was 33 cm when the arms were raised, leaving a space of 12.5 cm on each side to place the knee. Although no participants had lower limbs that were so thick that they were unable to straddle the patient, it is possible that when both the patient and the rescuer are of large build, there may not be sufficient space for the rescuer to straddle the patient. Furthermore, the stretcher load capacity and width in one’s institution should be checked since the combined weight may exceed its load capacity. Straddling CPR is advised against for some stretchers in their product instruction manuals. It should be kept in mind that one will be held responsible in cases of trouble such as damage to the stretcher.

### Limitations

As mentioned earlier, the greatest bias of this study was that the participants were not blinded to the two CPR methods. We believe that the difference between male participants and female participants is significant, but we cannot prove this owing to the serious occupational bias. Effectiveness in living individuals has yet to be studied. It is also unknown whether the results of the present study can be applied to patients or rescuers with a large build. Moreover, there are insufficient data to discuss adverse events such as falls, damage to the sternocostal junction and stretcher damage; thus, more cases must be collected. It was not possible to verify in this research what kind of differences would have arisen between the two groups in the absence of the metronome.

## Conclusion

Chest compression depth significantly improved in straddling CPR compared to walking CPR, whereas recoil, rate, and hand position were not affected. Female participants were unable to achieve a sufficient chest compression depth when performing walking CPR. This tendency became more marked over time. However, female participants were able to maintain a chest compression depth within the acceptable range during straddling CPR. Straddling CPR should be considered for female rescuers. On the other hand, walking CPR is acceptable if performed by male rescuers.

## Supporting information

S1 Table Data of chest compressions every time (raw data obtained from SimPad). Yellow coloured tabs indicate pretest, blue coloured tabs indicate straddling CPR, and red coloured tabs indicate pretest. Arabic numerals on the tabs indicate the participants’ numbers; the letter ‘P’ indicates pretest, the letter ‘S’ indicates straddling CPR, and the letter ‘W’ indicates walking CPR.
(XLSX)

S2 Table Data shown for each participants. (main data). (XLSX)

S1 Fig Waveform of chest compressions (depth and rate). (PDF)
