# Supplementary material for: Comparison of chest compression quality in walking versus straddling cardiopulmonary resuscitation during stretcher transportation: A prospective randomised crossover study using manikins
Source: PLoS One. 2019 May 21;14(5):e0216739. doi: 10.1371/journal.pone.0216739 (PMC6528974; doi:10.1371/journal.pone.0216739)

Participant ID: 1, Height 173cm, Weight 60kg, BMI 20kg/cm, age: 20s, sex: male

walking CPR

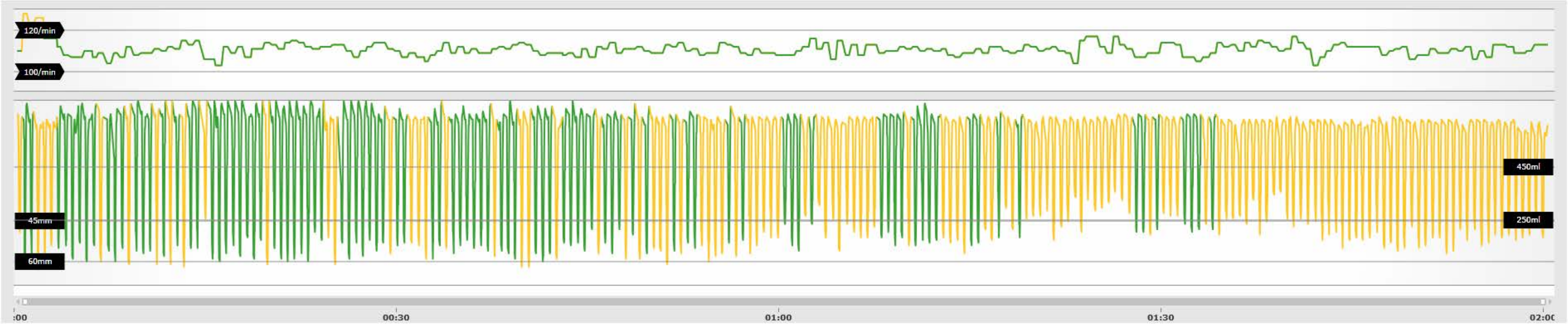

straddling CPR

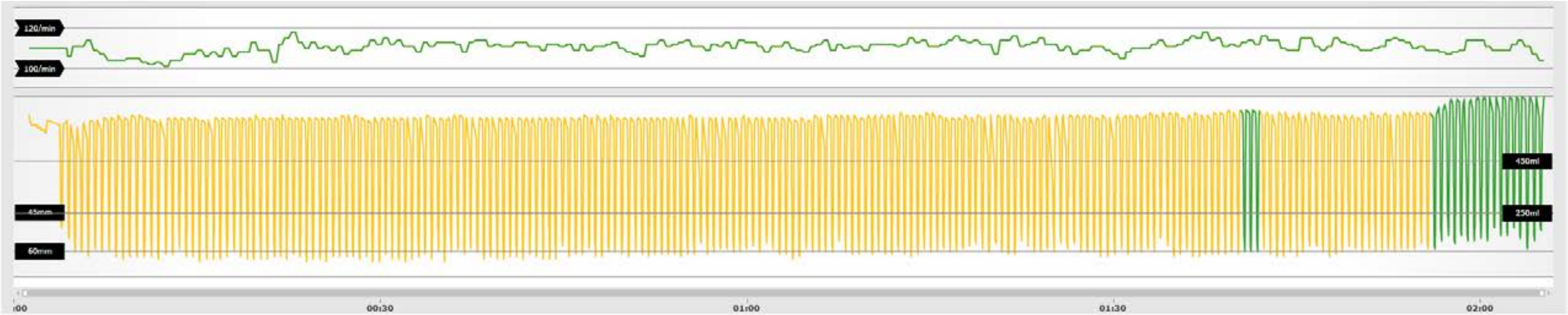

pretest

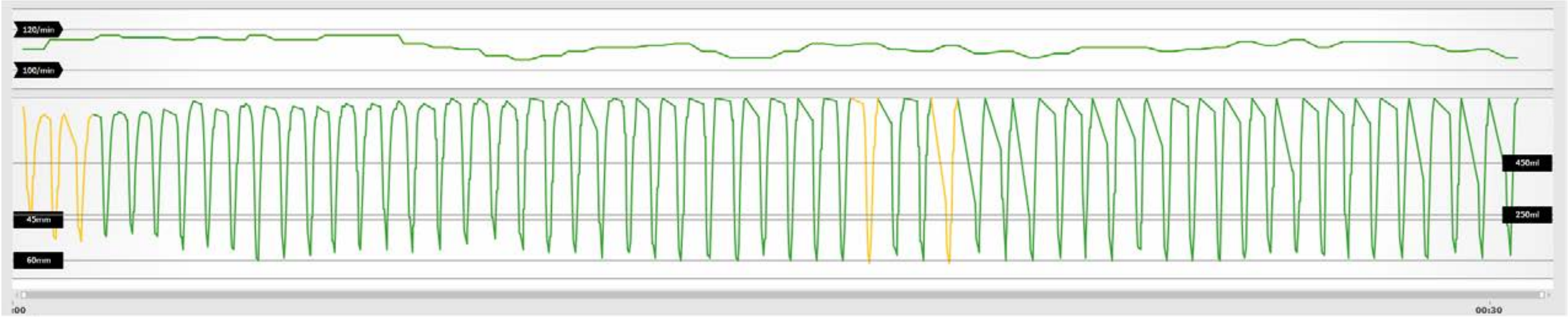

Participant ID: 2, Height 166cm, Weight 66kg, BMI 24kg/cm, age: 30s, sex: male

walking CPR

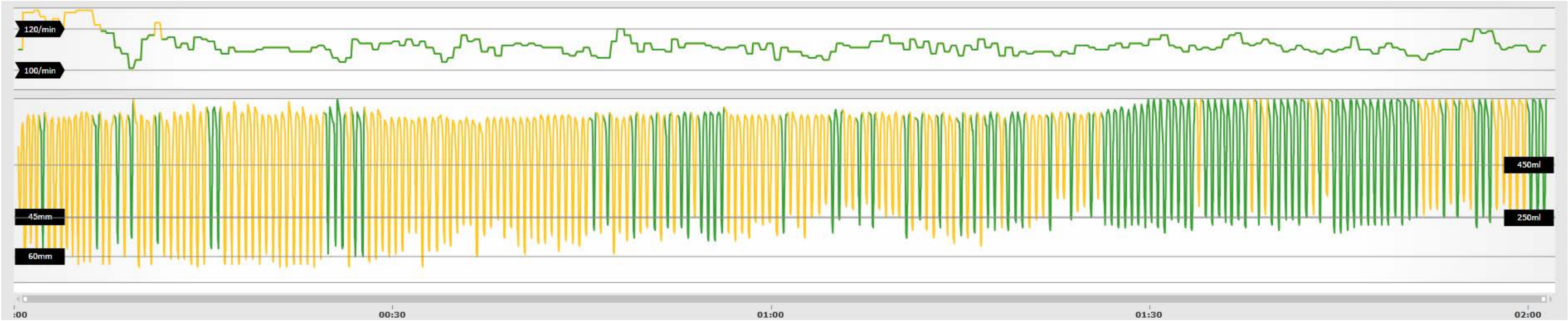

straddling CPR

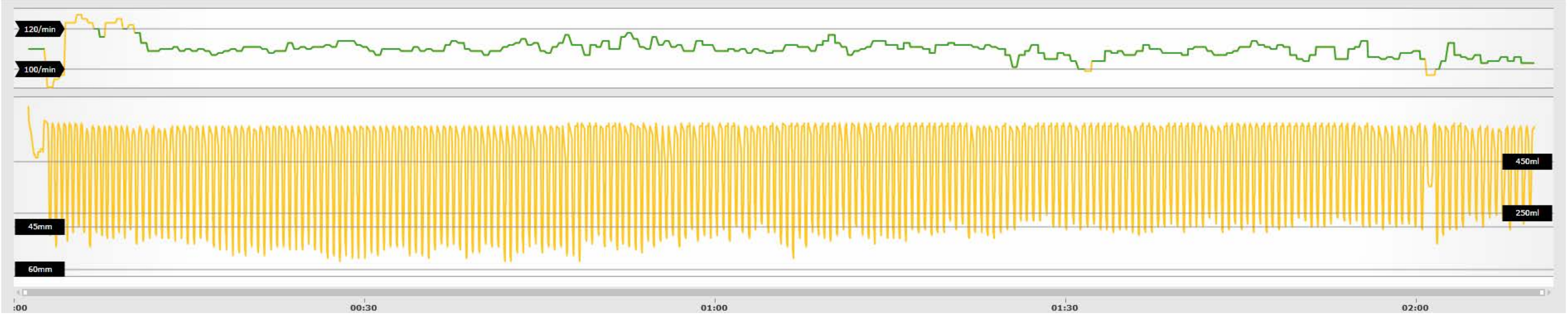

pretest

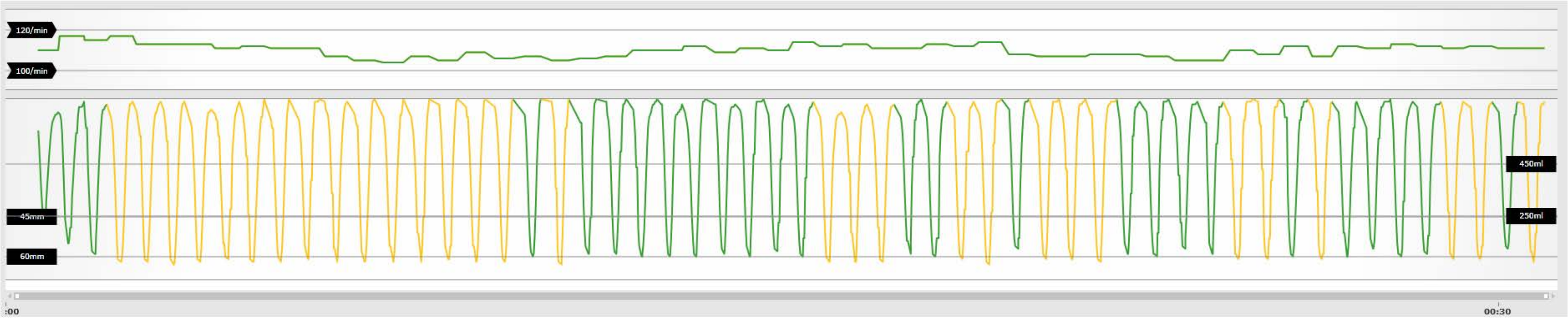

Participant ID: 3,   Height 155cm,   Weight 47kg,   BMI 20kg/cm,   age: 40s,   sex: female

walking CPR

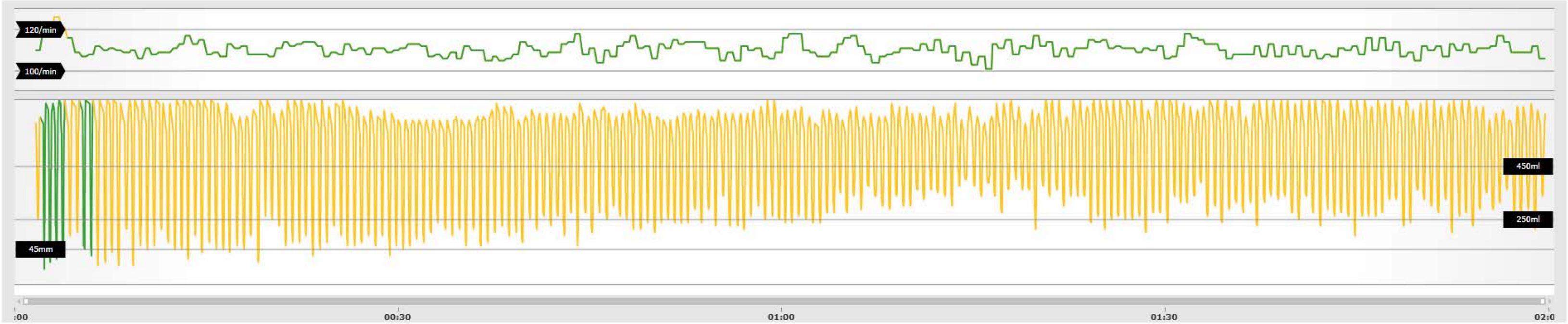

straddling CPR

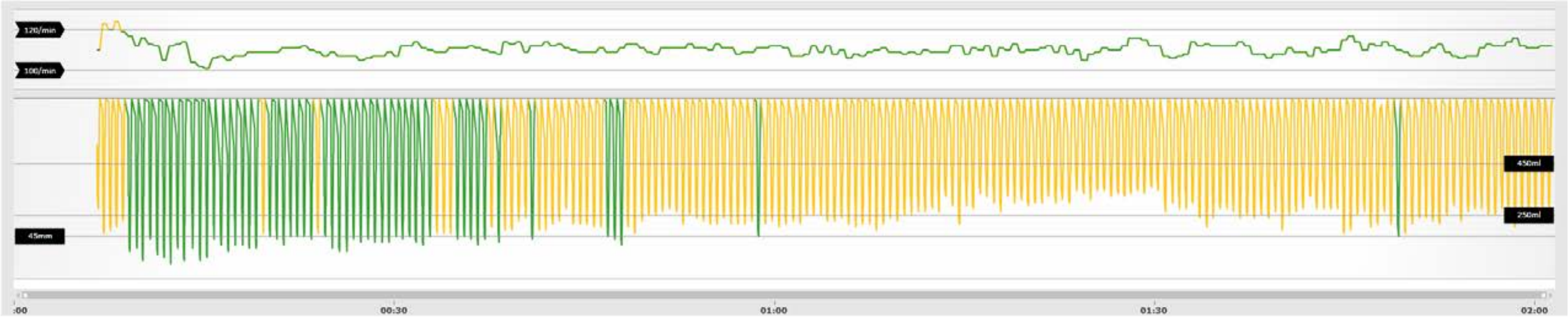

pretest

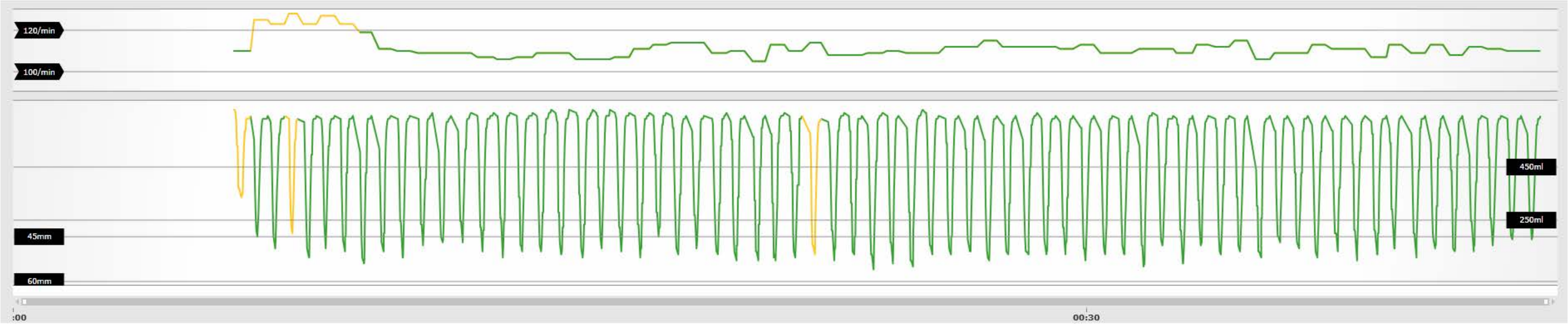

Participant ID: 4,   Height 150cm,   Weight 58kg,   BMI 26kg/cm,   age: 30s,   sex: female

walking CPR

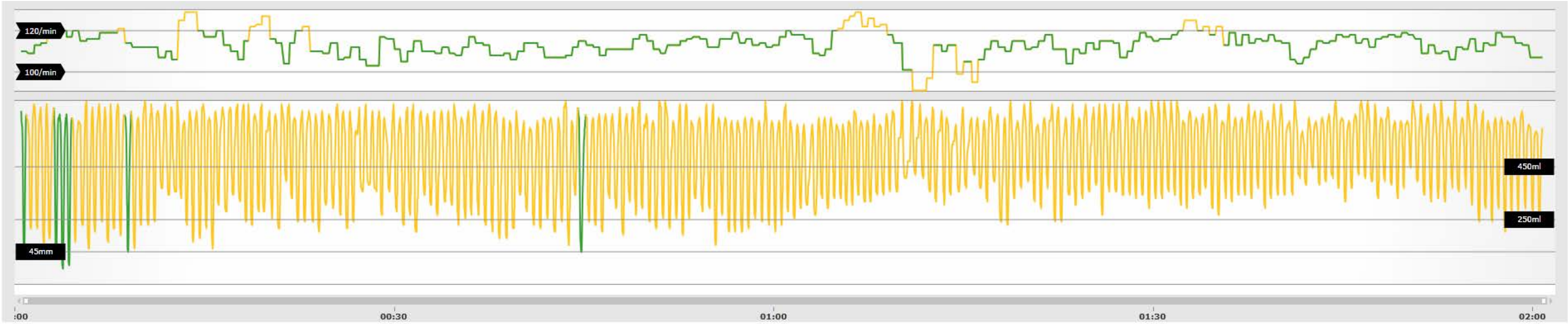

straddling CPR

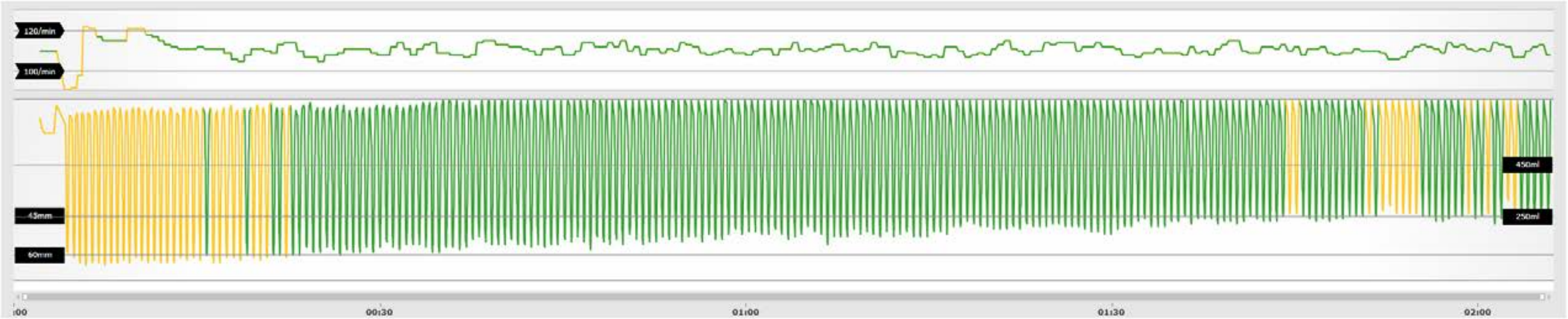

pretest

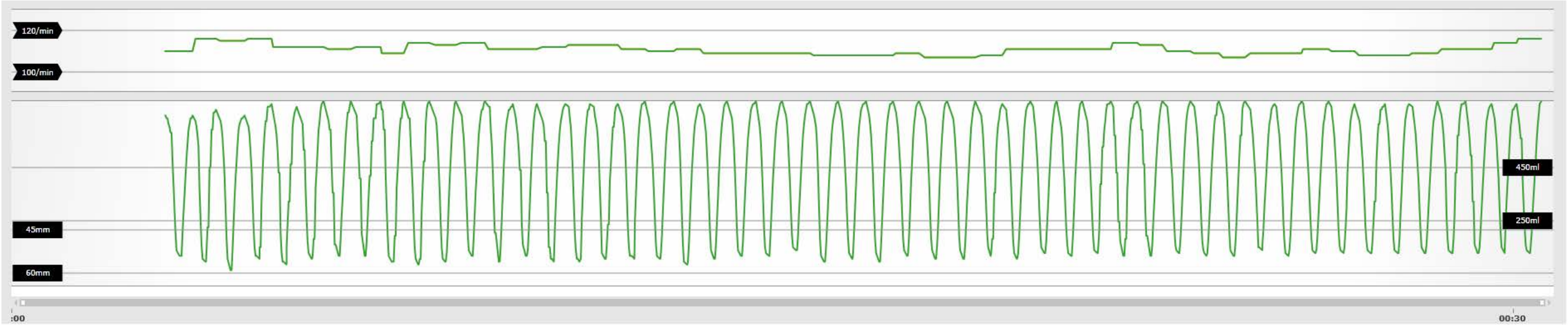

Participant ID: 5, Height 163cm, Weight 52kg, BMI 20kg/cm, age: 30s, sex: female

walking CPR

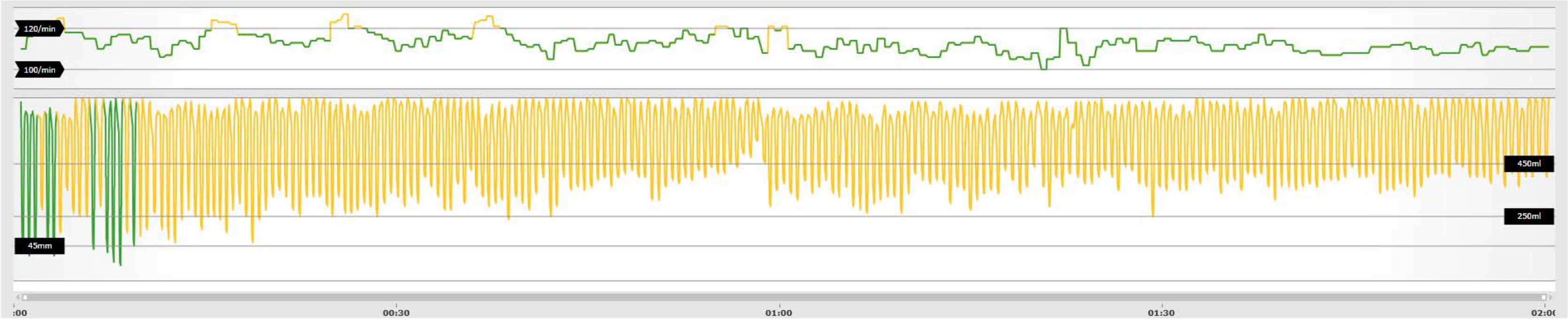

straddling CPR

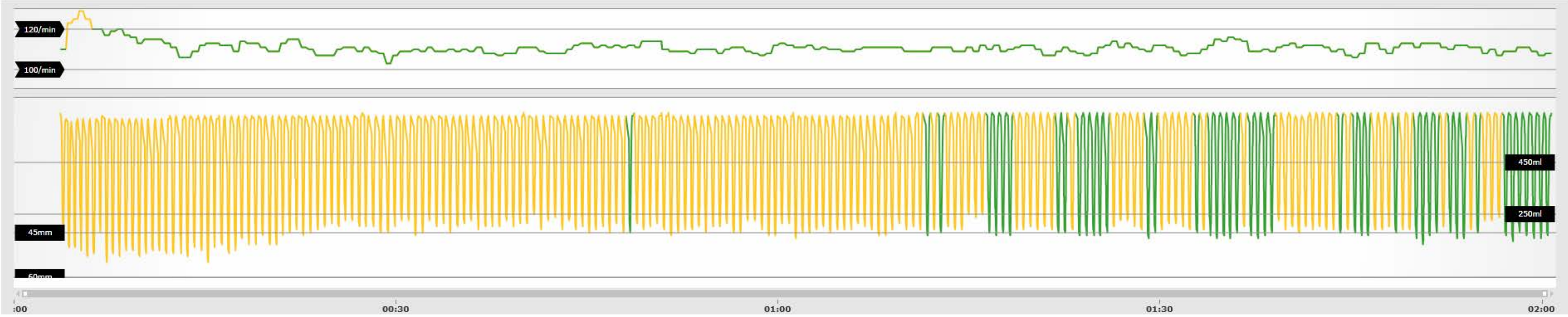

pretest

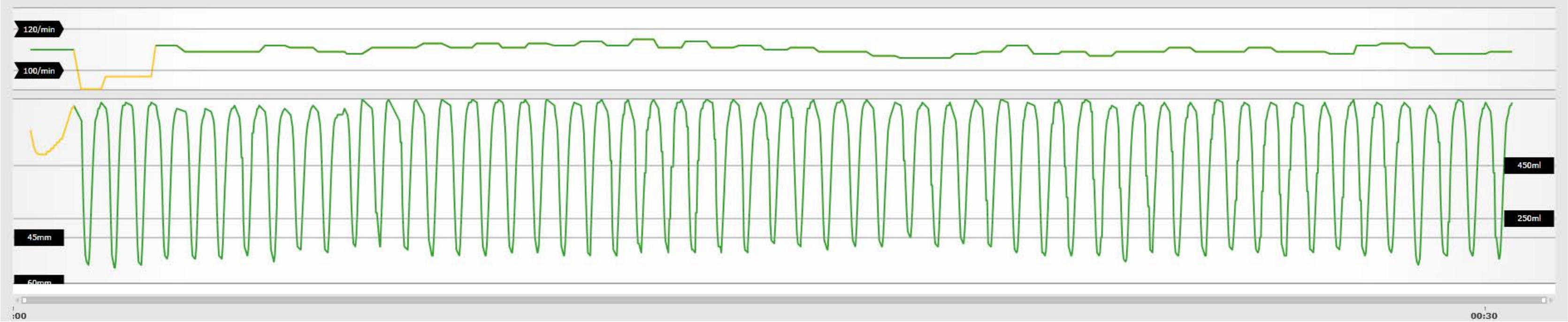

Participant ID: 6, Height 173cm, Weight 68kg, BMI 23kg/cm, age: 20s, sex: male

walking CPR

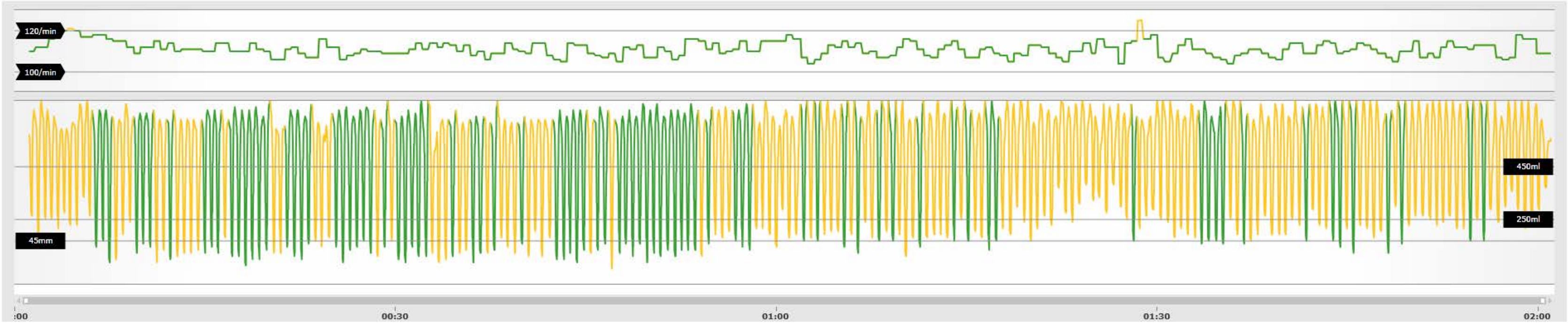

straddling CPR

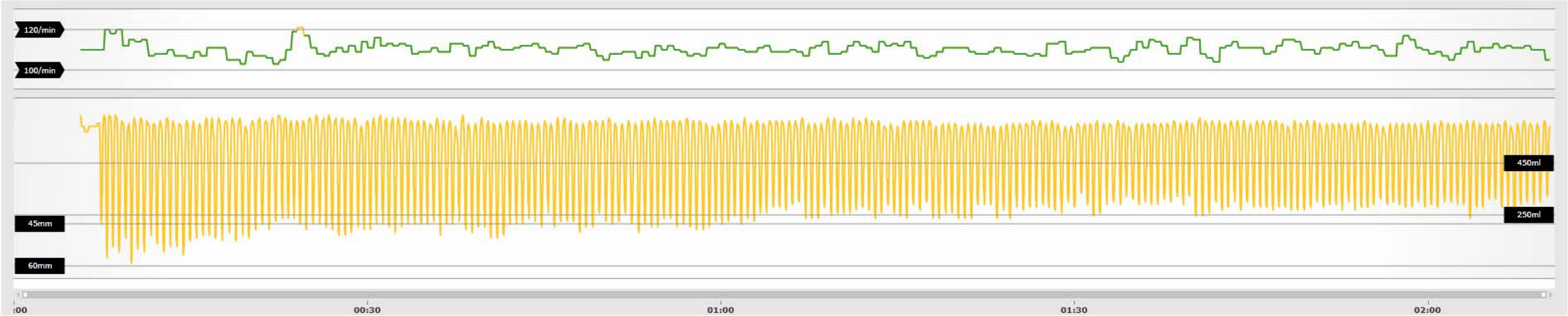

pretest

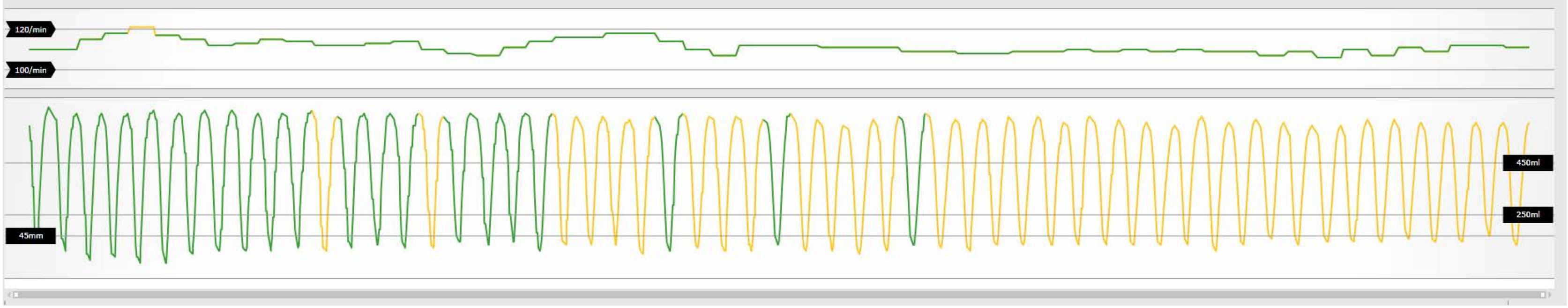

Participant ID: 7,   Height 187cm,   Weight 69kg,   BMI 20kg/cm,   age: 40s,   sex: male

walking CPR

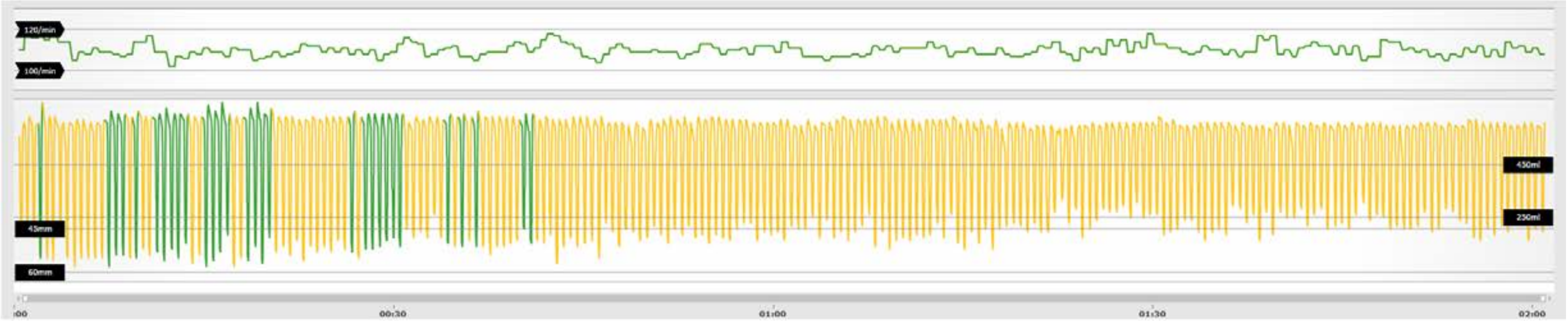

straddling CPR

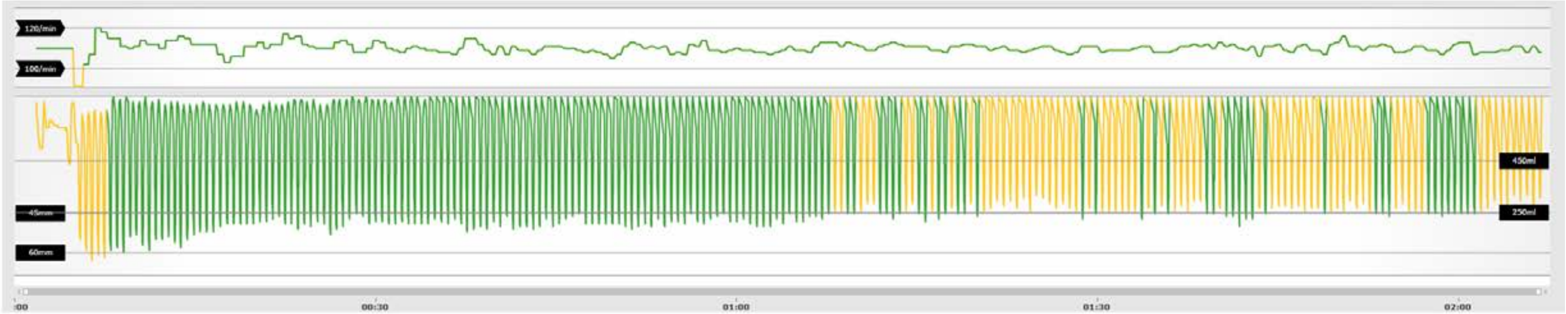

pretest

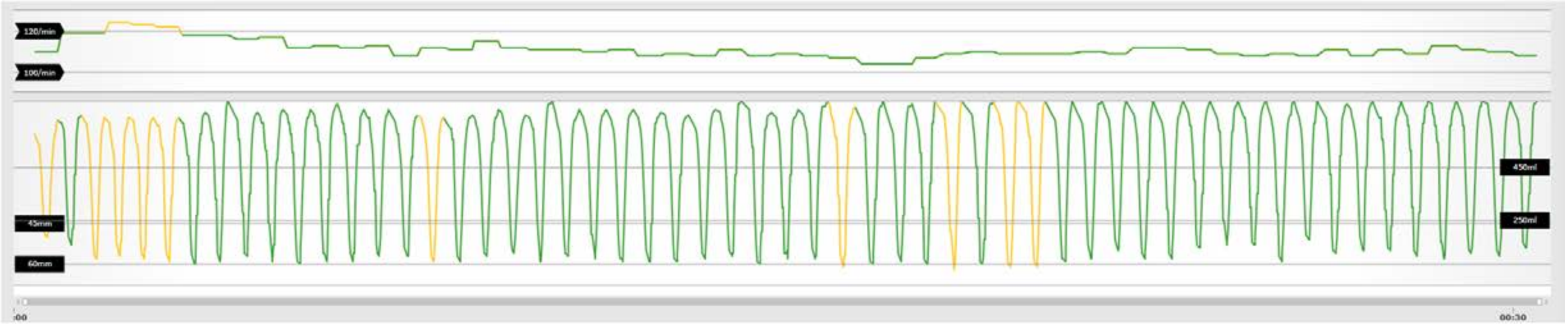

Participant ID: 8,   Height 159cm,   Weight 56kg,   BMI 22kg/cm,   age: 40s,   sex: female

walking CPR

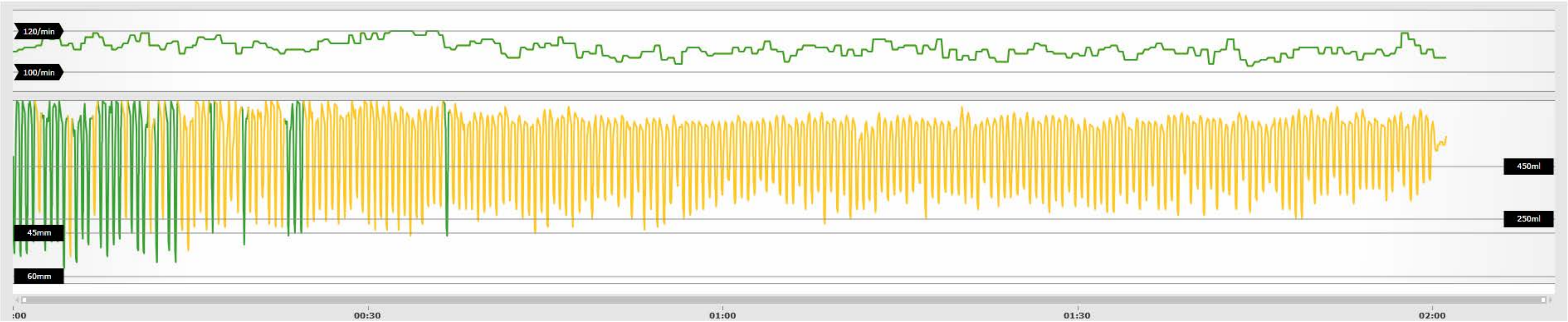

straddling CPR

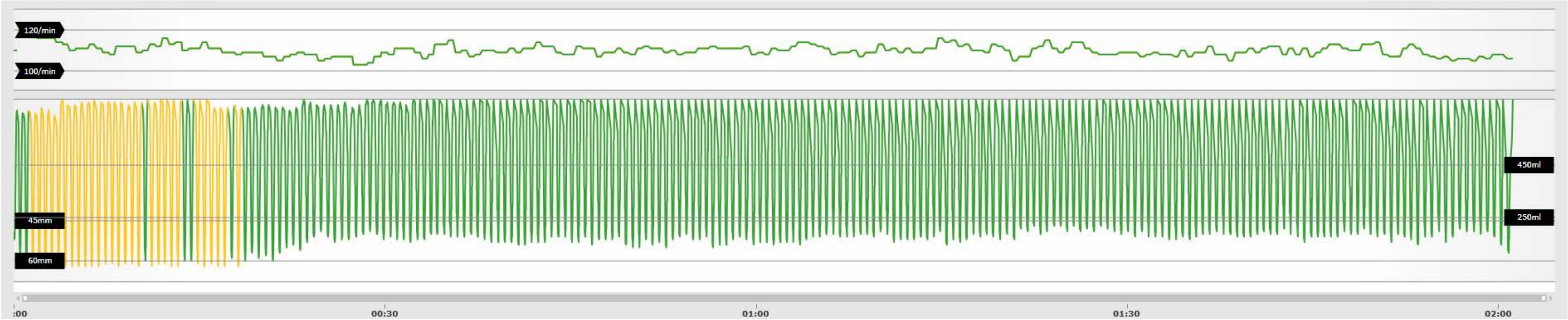

pretest

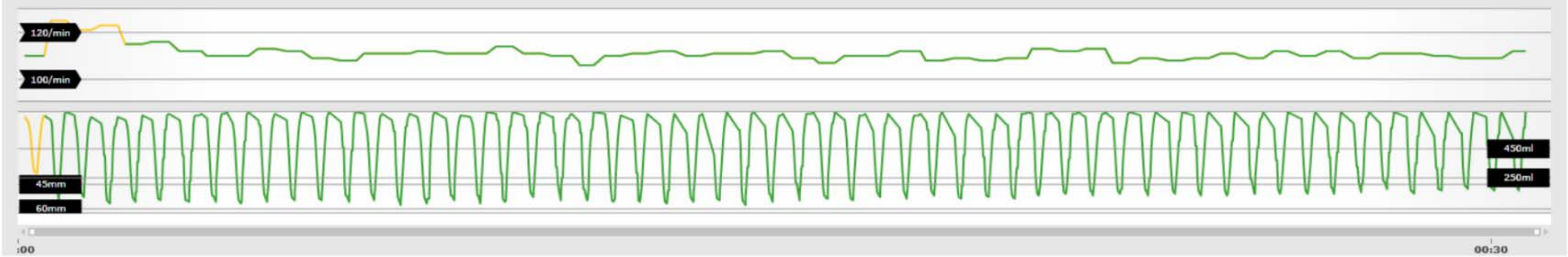

Participant ID: 9,   Height 157cm,   Weight 52kg,   BMI 21kg/cm,   age: 30s,   sex: female

walking CPR

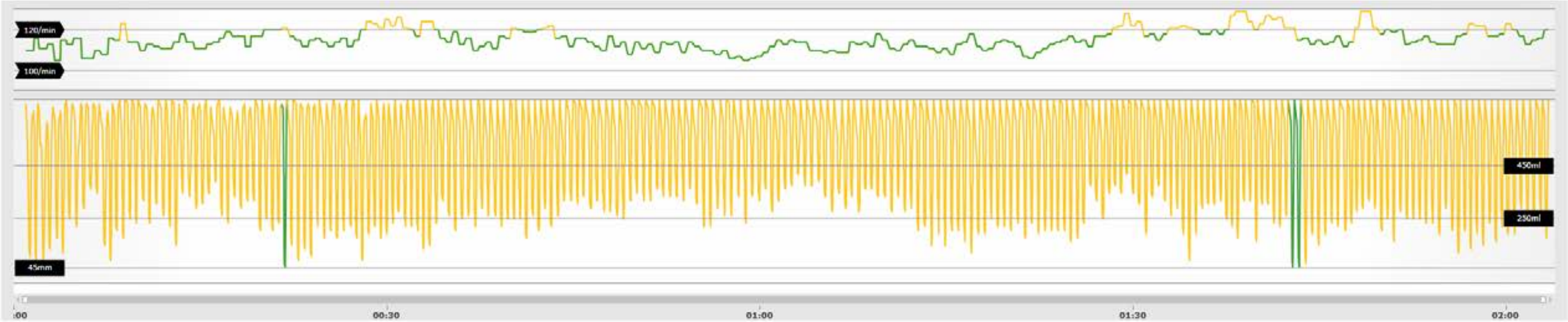

straddling CPR

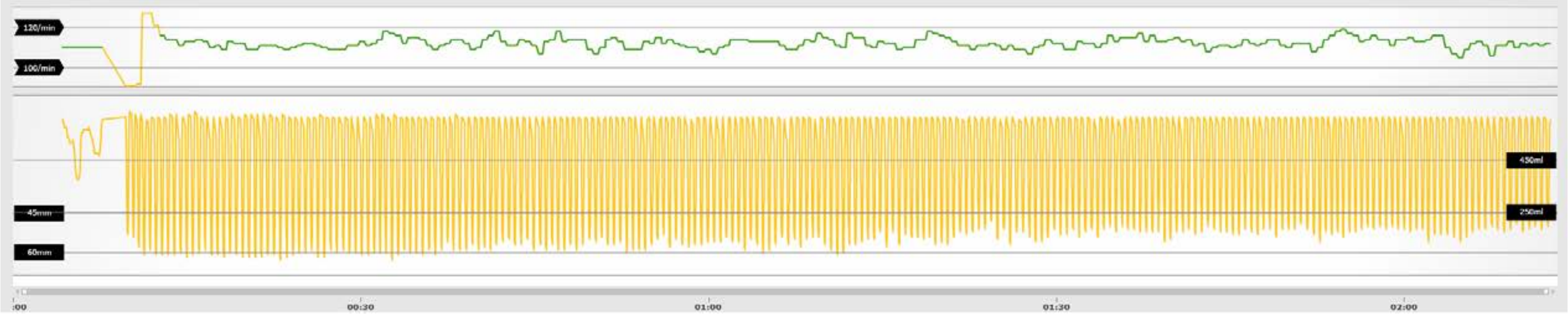

pretest

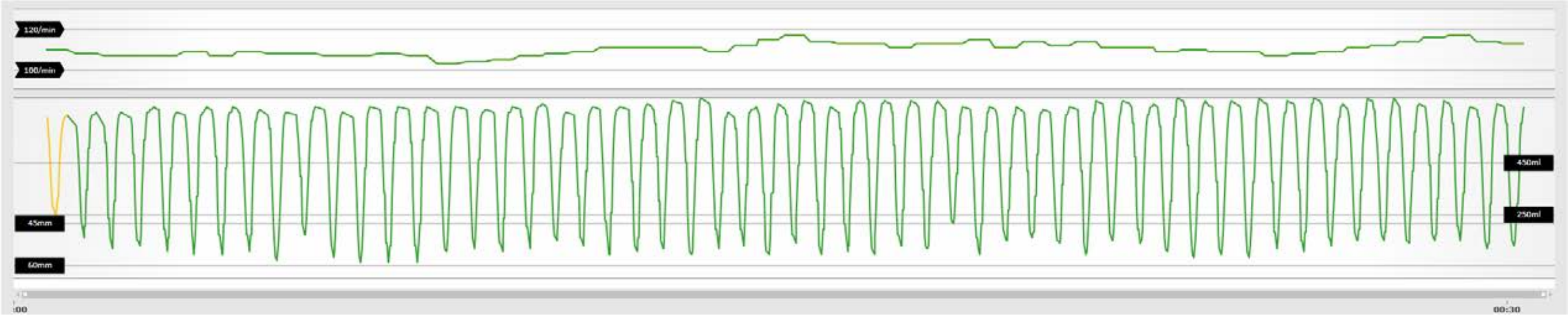

Participant ID: 10,   Height 183cm,   Weight 78kg,   BMI 23kg/cm,   age: 20s,   sex: male

walking CPR

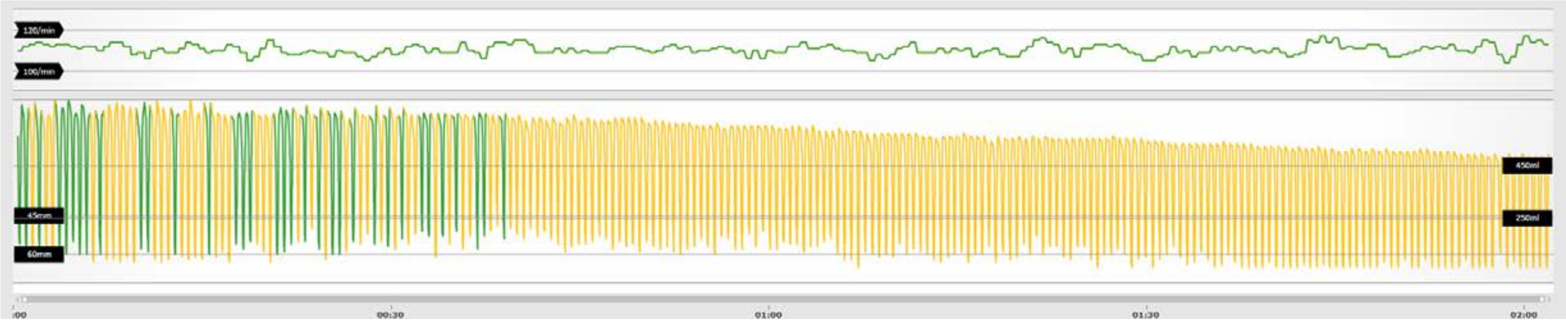

straddling CPR

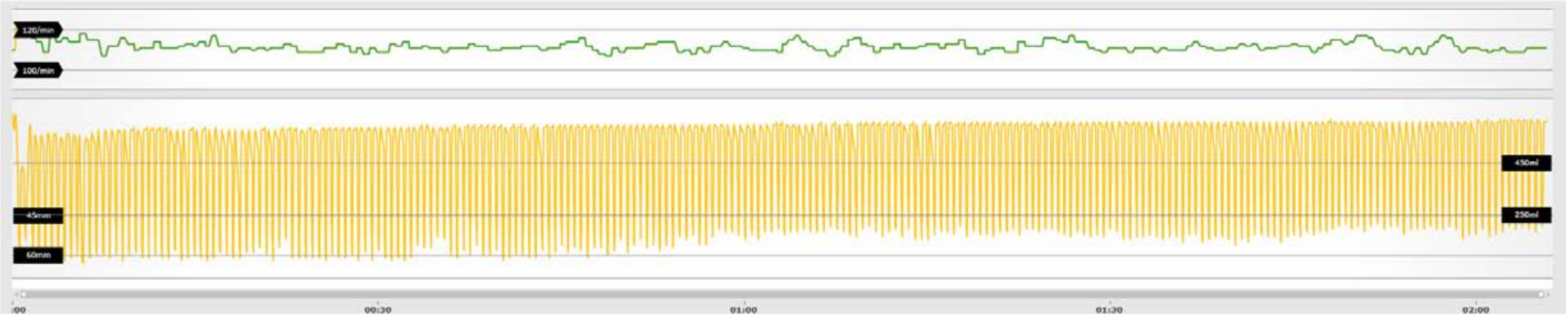

pretest

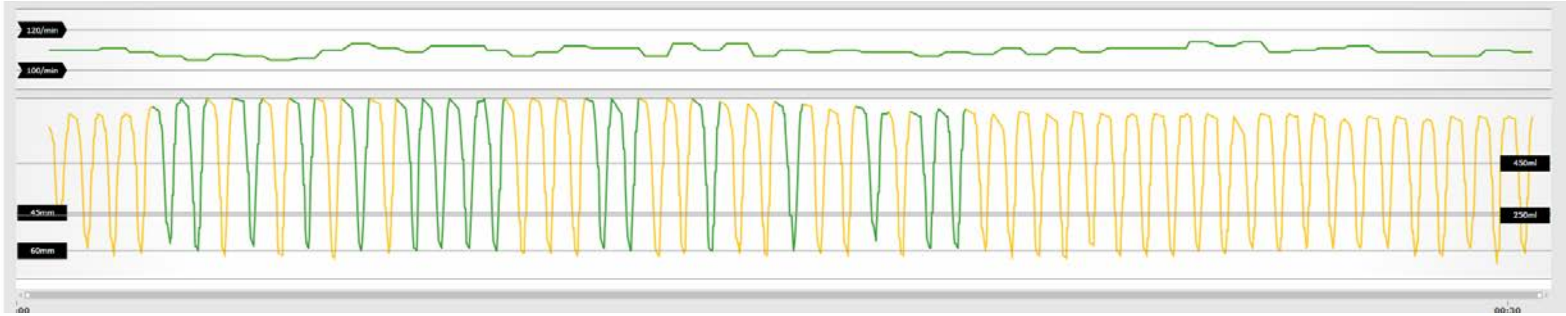

Participant ID: 11, Height 167cm, Weight 60kg, BMI 22kg/cm, age: 60s, sex: male

walking CPR

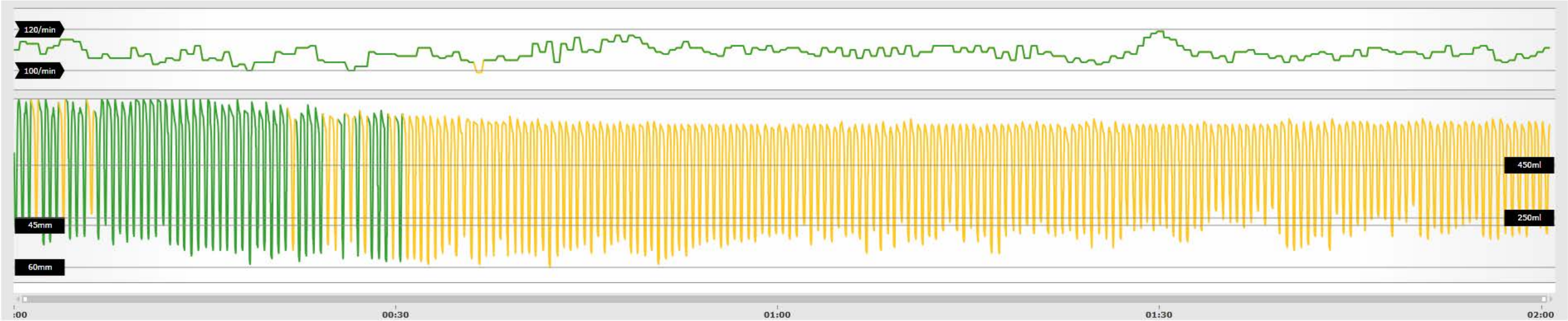

straddling CPR

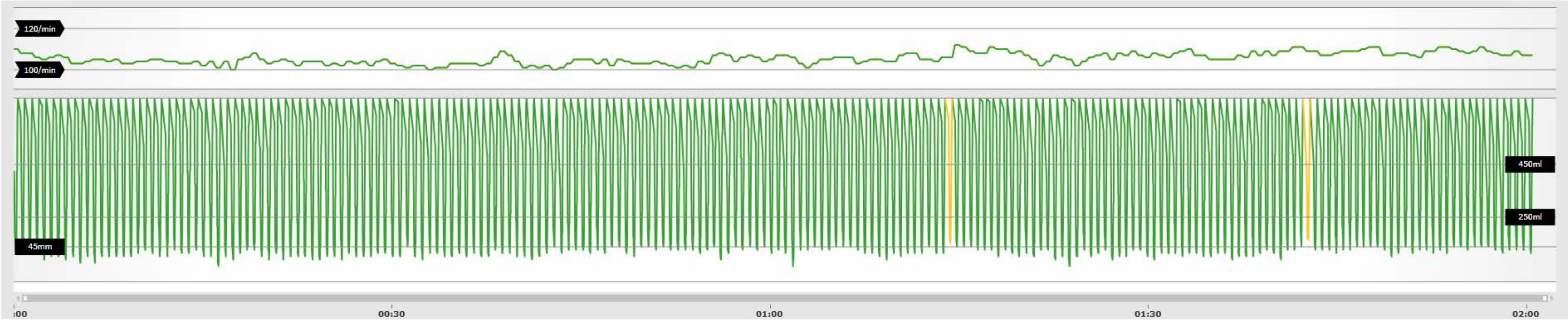

pretest

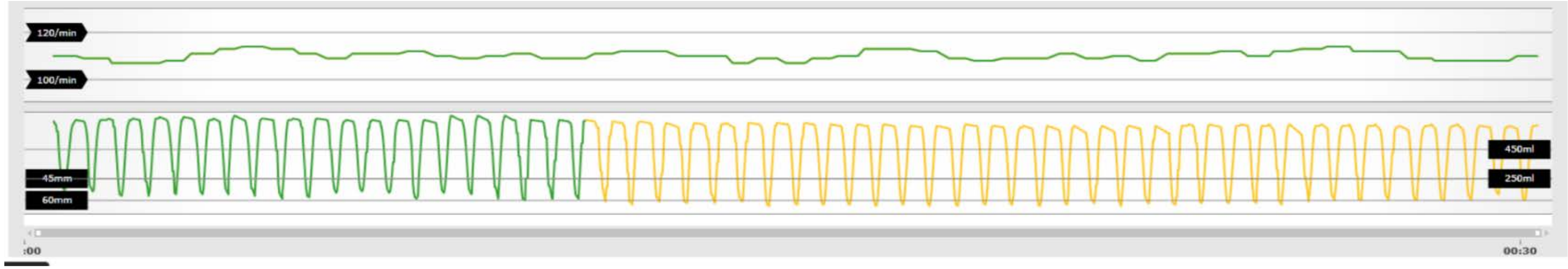

Participant ID: 12, Height 165cm, Weight 58kg, BMI 21kg/cm, age: 30s, sex: male

walking CPR

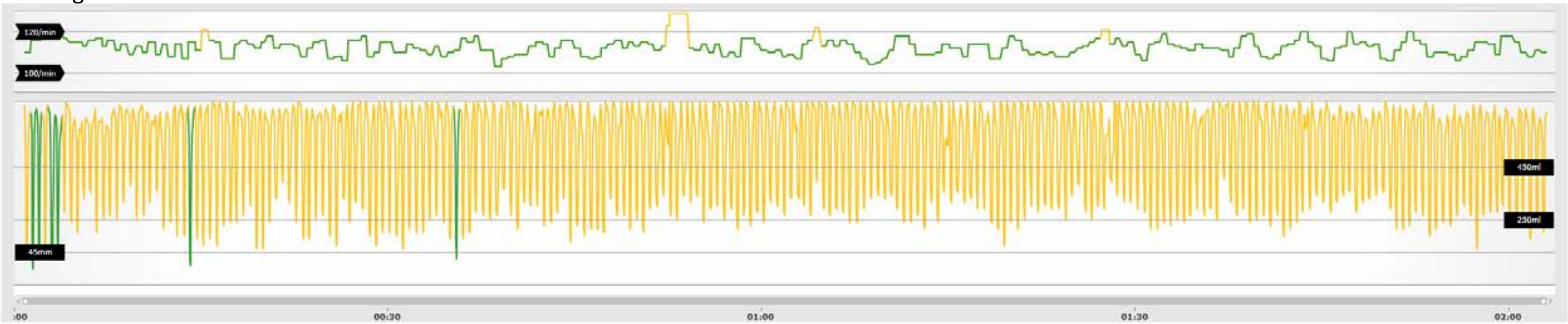

straddling CPR

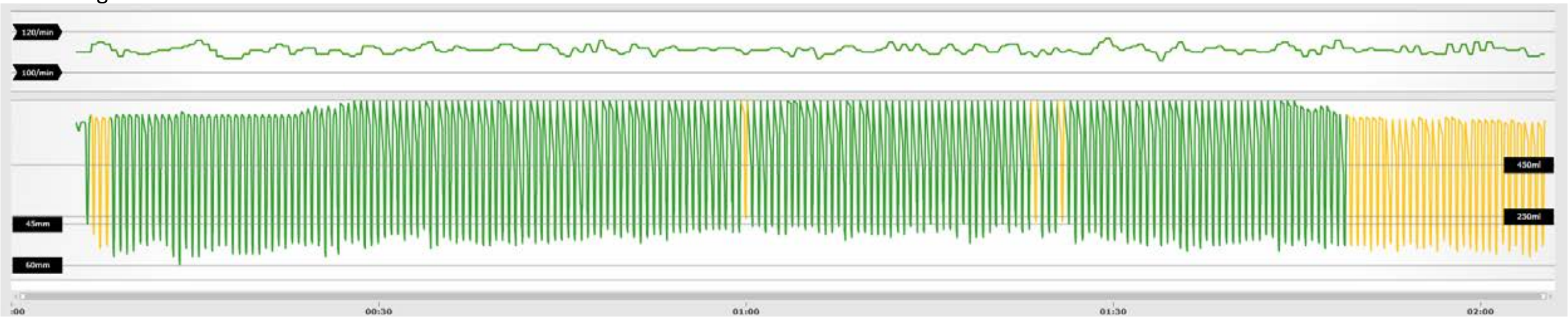

pretest

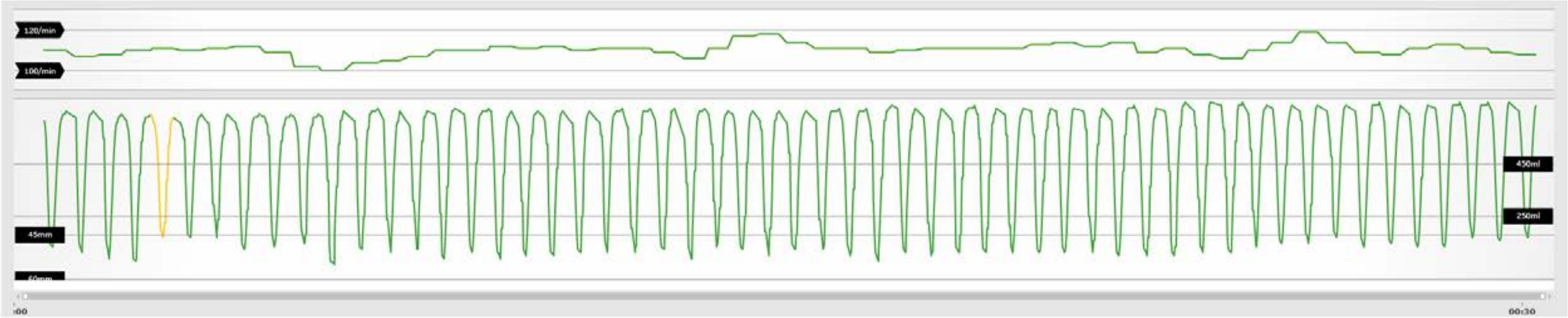

Participant ID: 13, Height 159cm, Weight 52kg, BMI 21kg/cm, age: 30s, sex: female

walking CPR

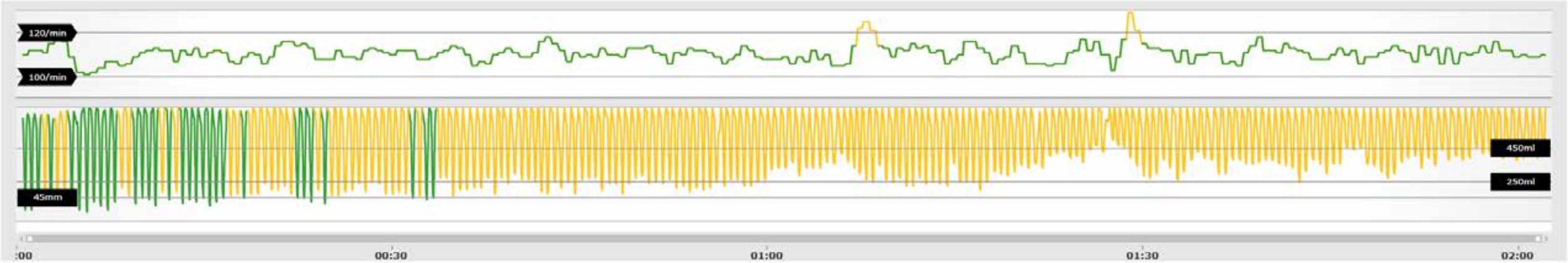

straddling CPR

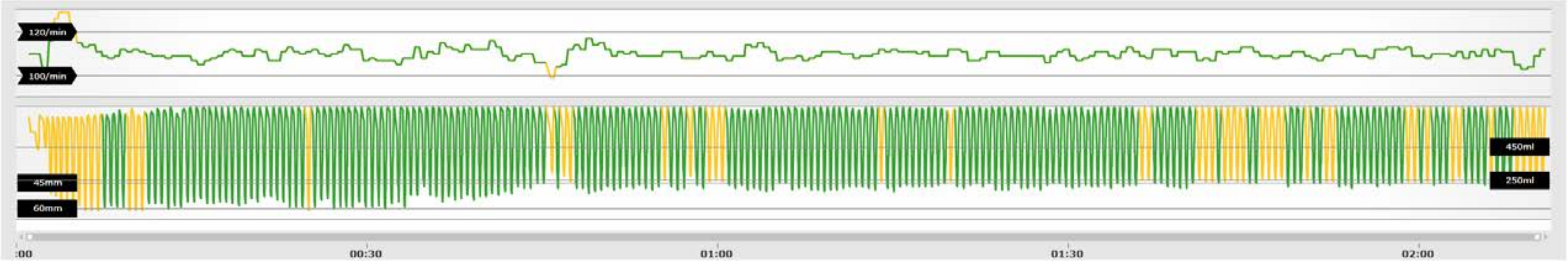

pretest

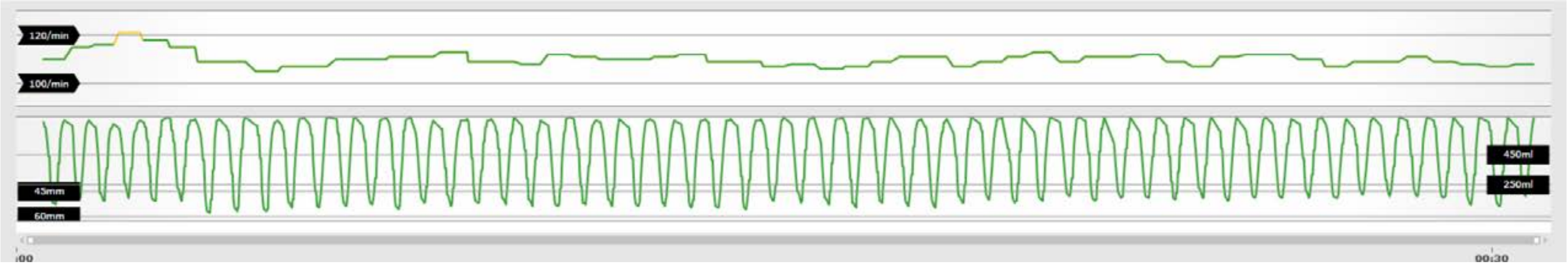

Participant ID: 14, Height 154cm, Weight 68kg, BMI 29kg/cm, age: 40s, sex: female

walking CPR

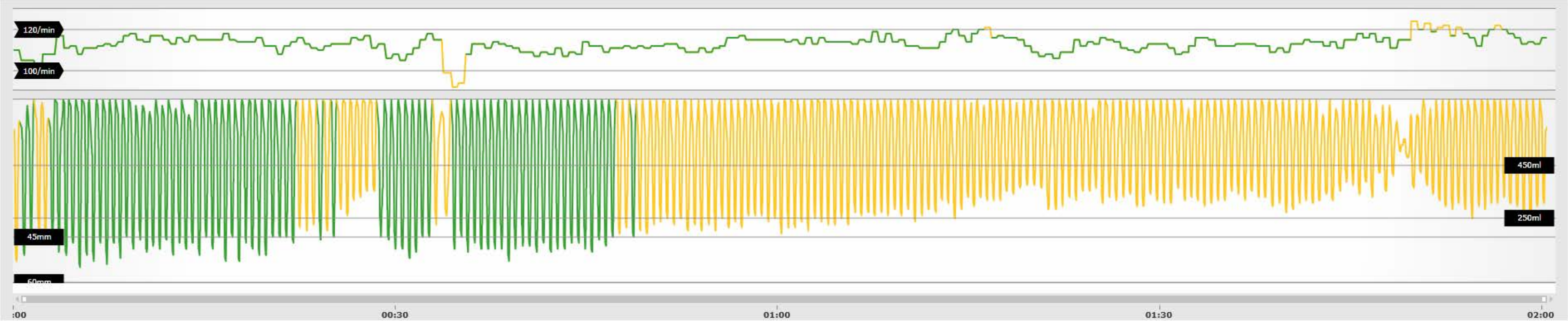

straddling CPR

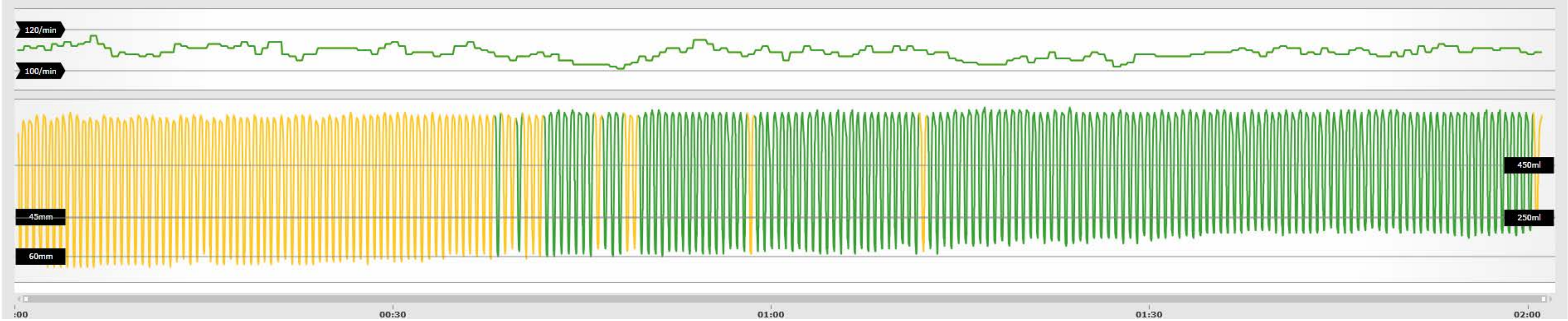

pretest

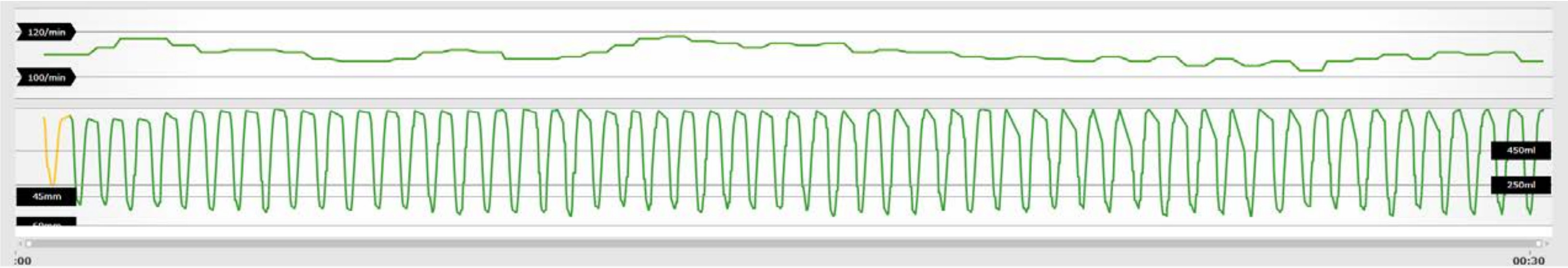

Participant ID: 15,   Height 174cm,   Weight 74kg,   BMI 24kg/cm,   age: 20s,   sex: male

walking CPR

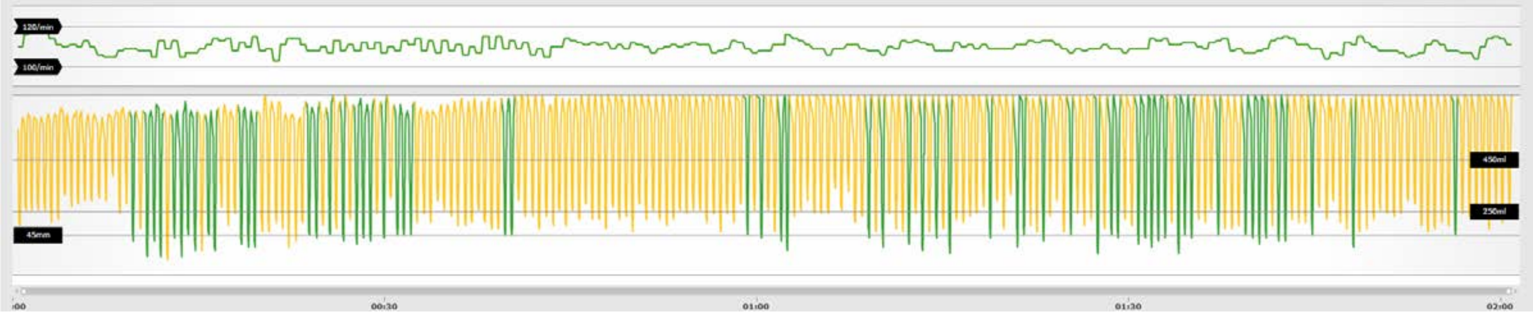

straddling CPR

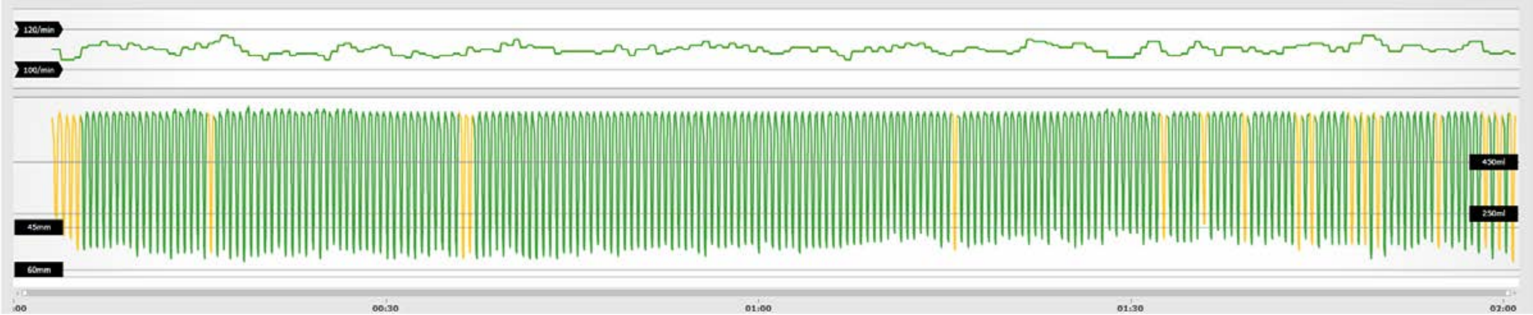

pretest

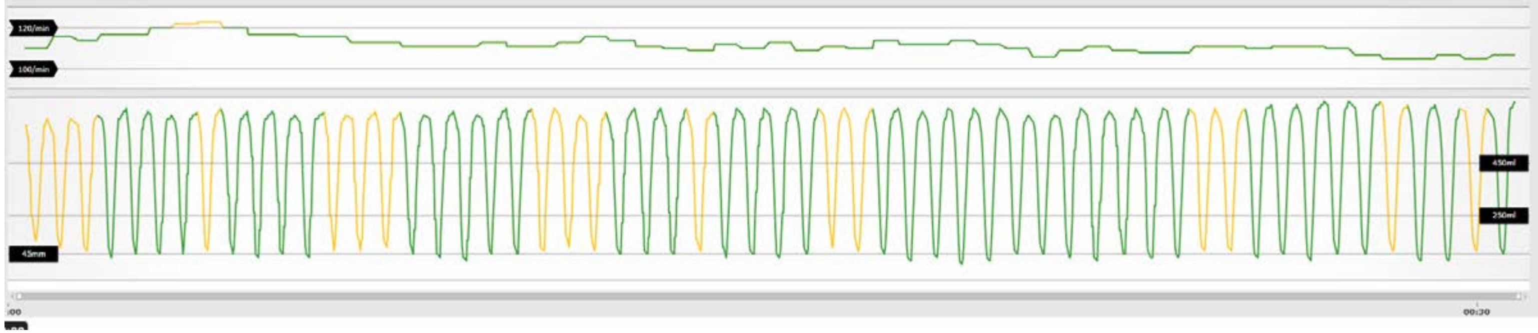

Participant ID: 16,   Height 162cm,   Weight 67kg,   BMI 26kg/cm,   age: 40s,   sex: female

walking CPR

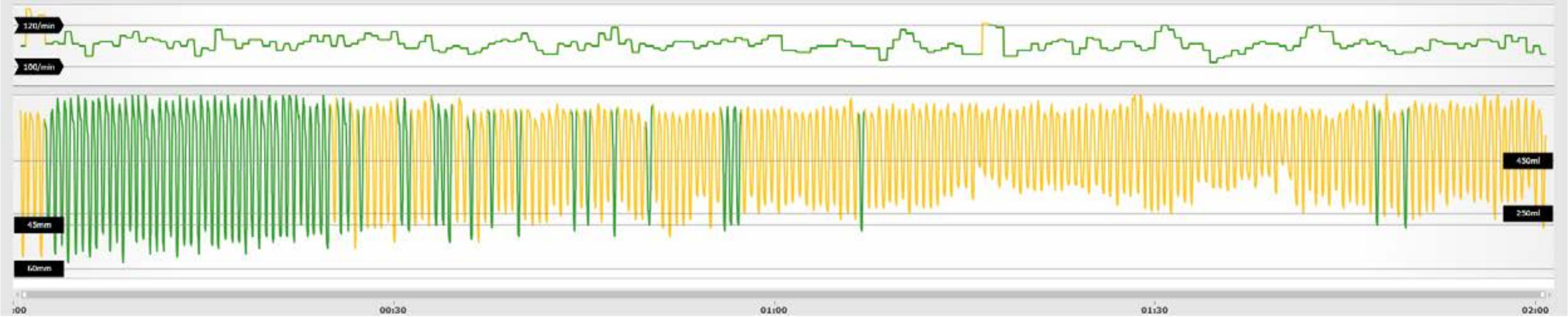

straddling CPR

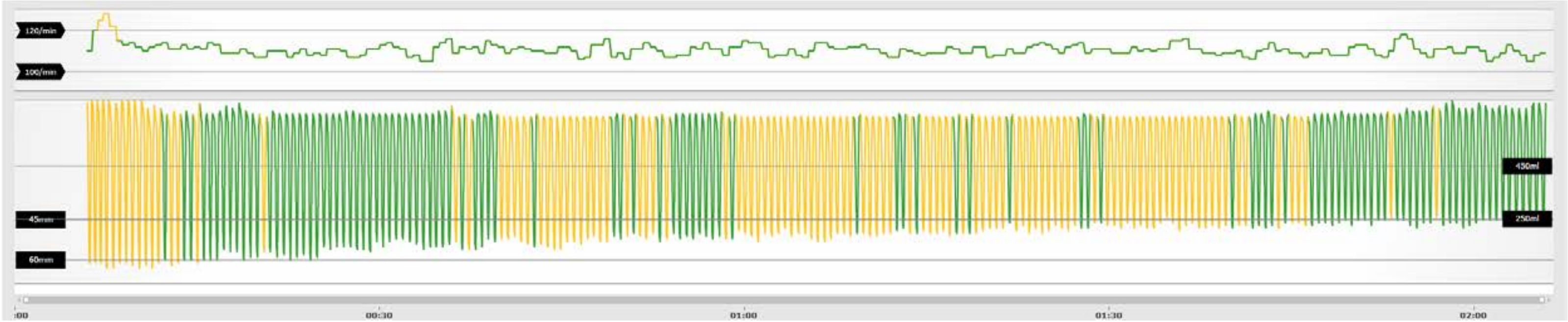

pretest

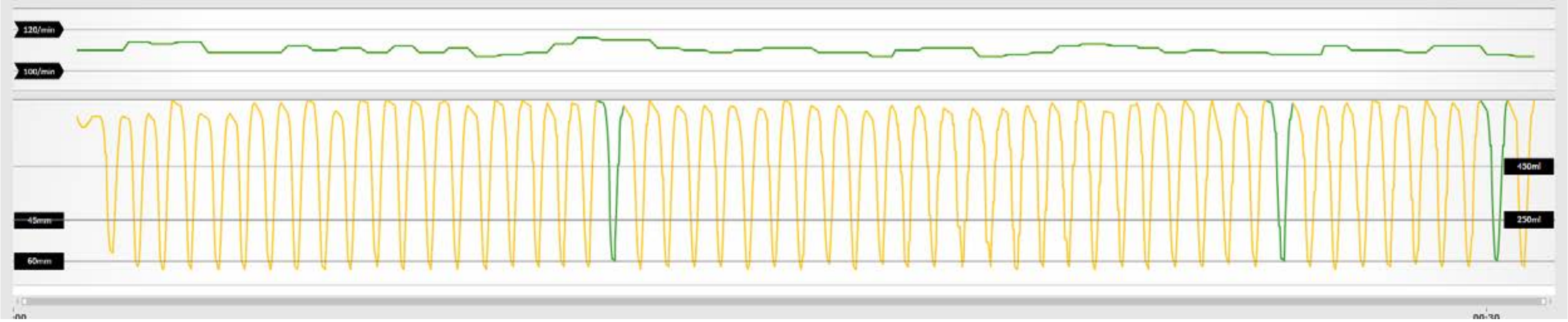

Participant ID: 17,   Height 180cm,   Weight 75kg,   BMI 23kg/cm,   age: 30s,   sex: male

walking CPR

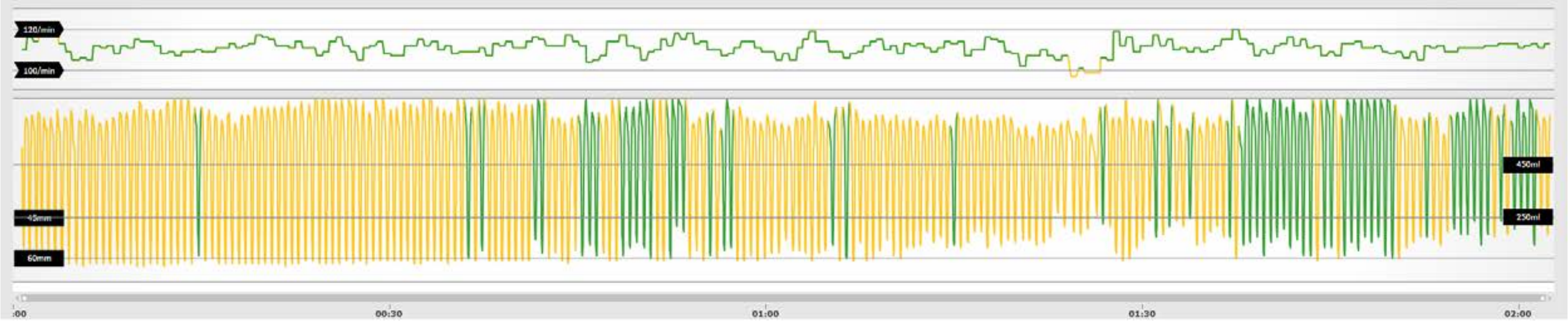

straddling CPR

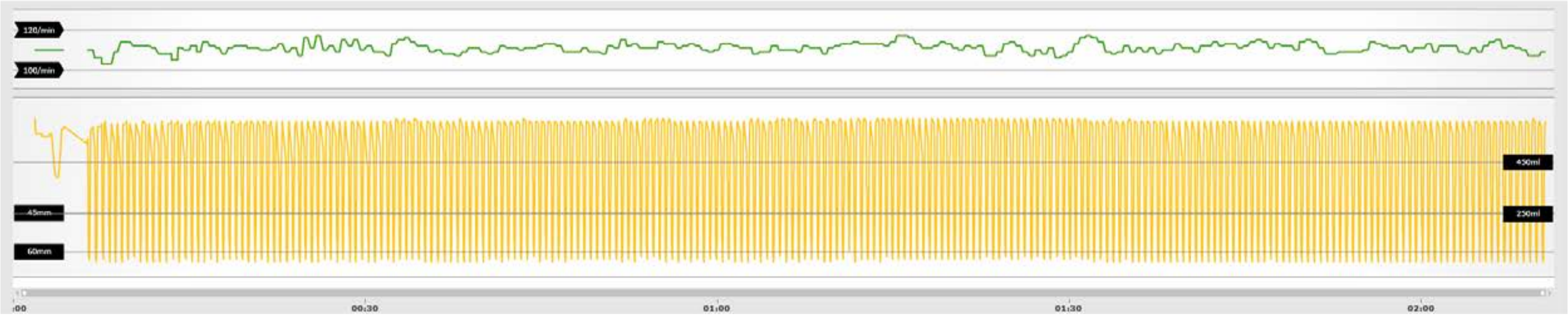

pretest

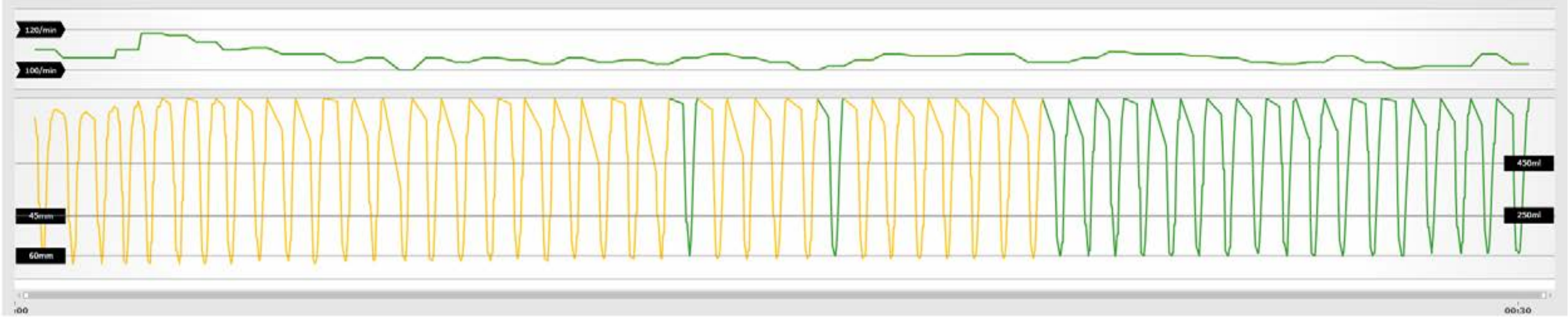

Participant ID: 18, Height 158cm, Weight 62kg, BMI 25kg/cm, age: 50s, sex: female

walking CPR

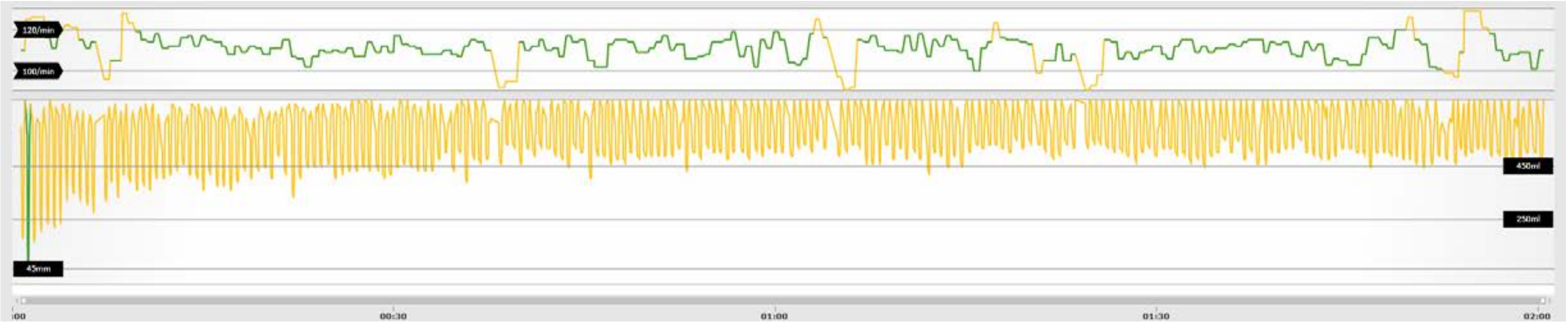

straddling CPR

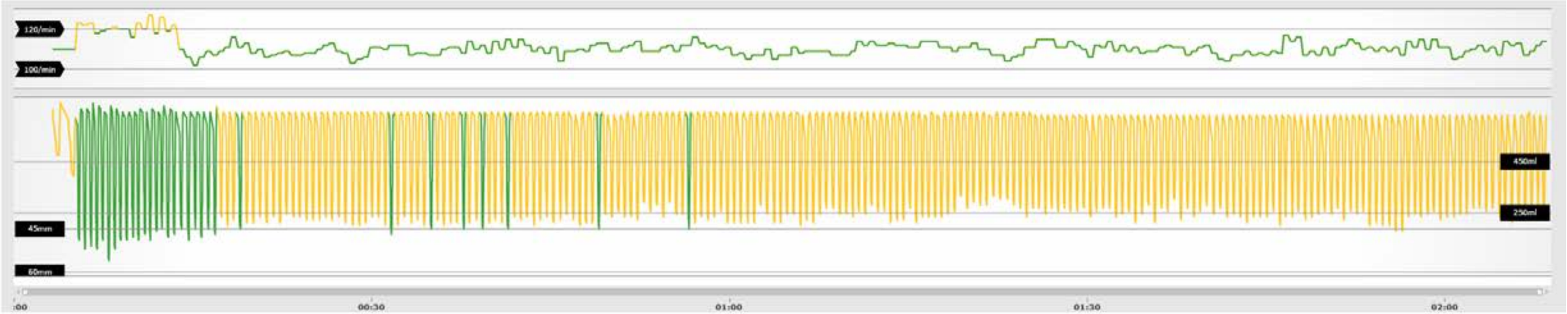

pretest

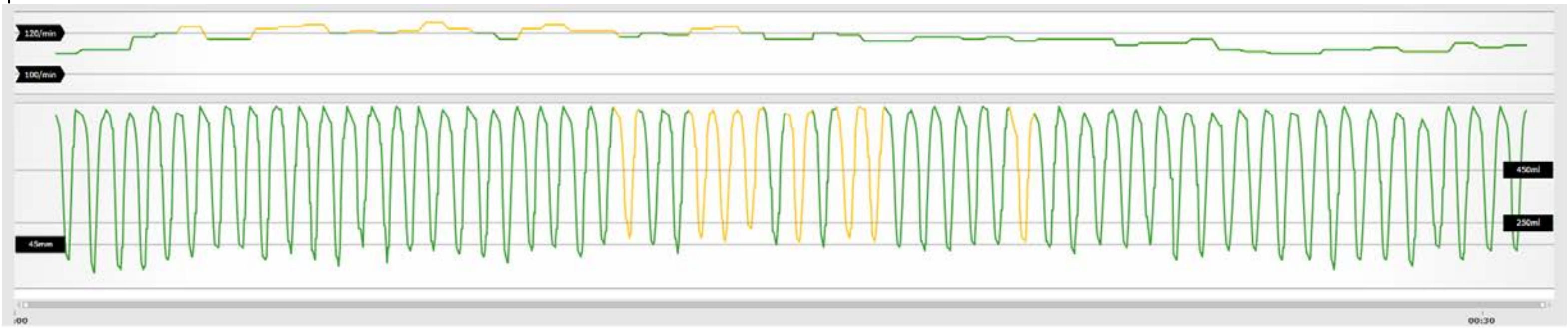

Participant ID: 19, Height 170cm, Weight 70kg, BMI 24kg/cm, age: 20s, sex: male

walking CPR

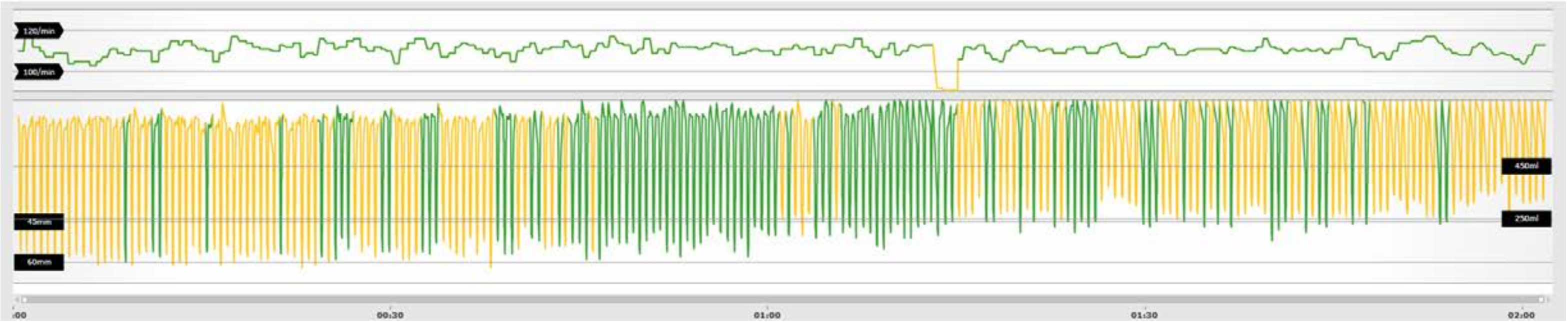

straddling CPR

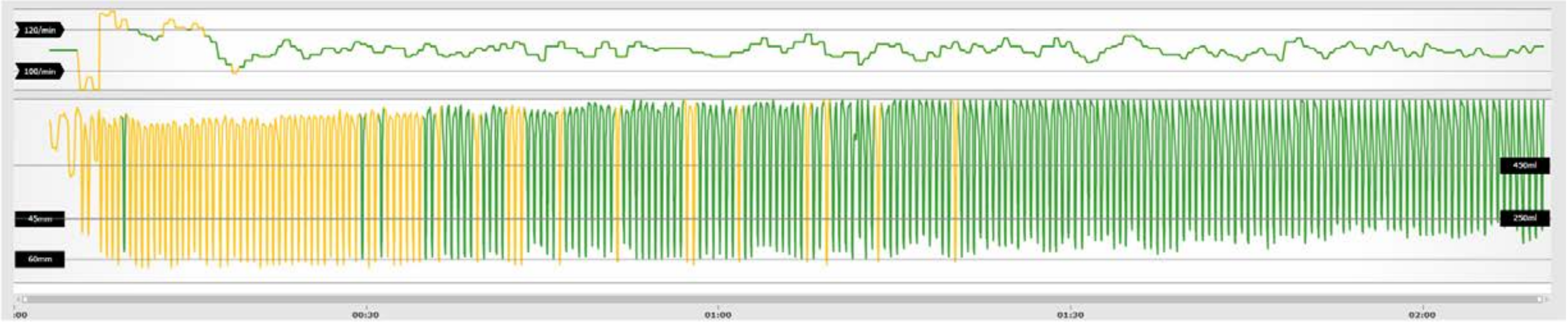

pretest

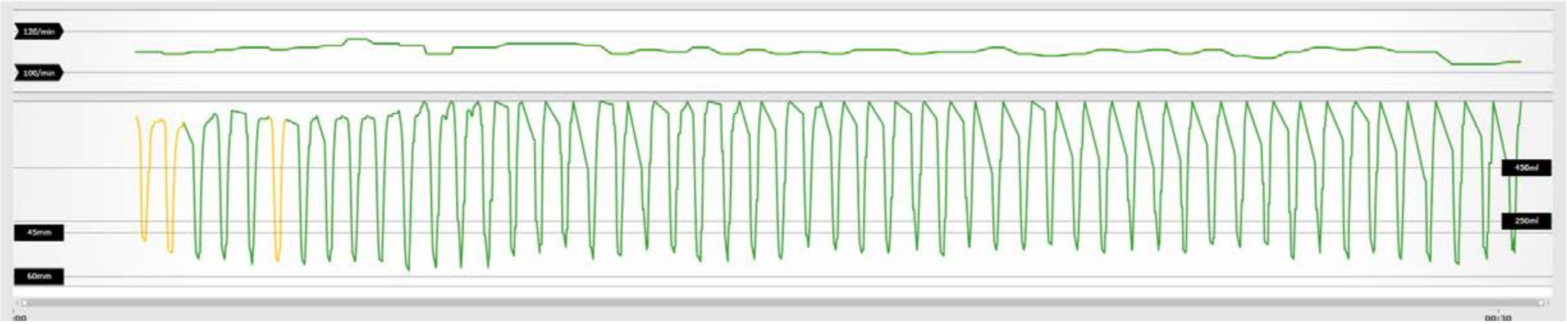

Participant ID: 20,   Height 155cm,   Weight 68kg,   BMI 28kg/cm,   age: 30s,   sex: female

walking CPR

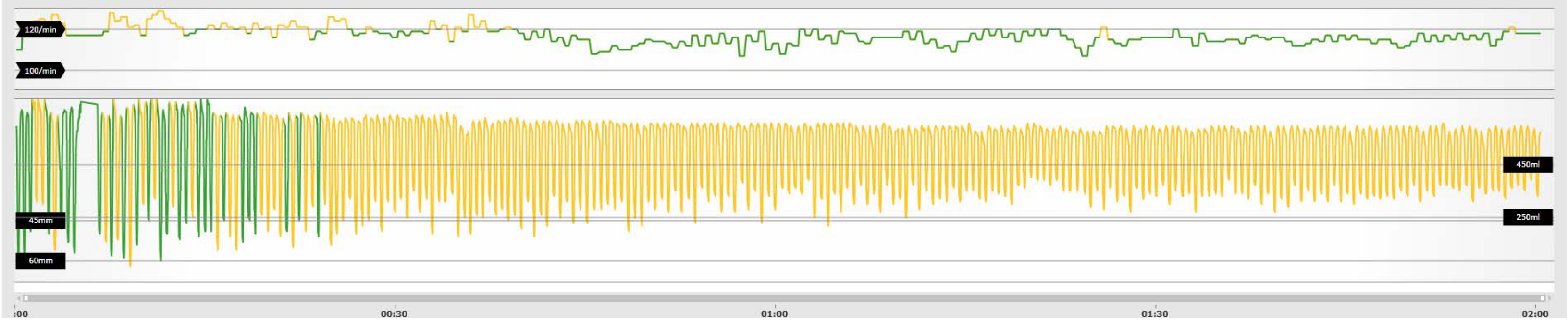

straddling CPR

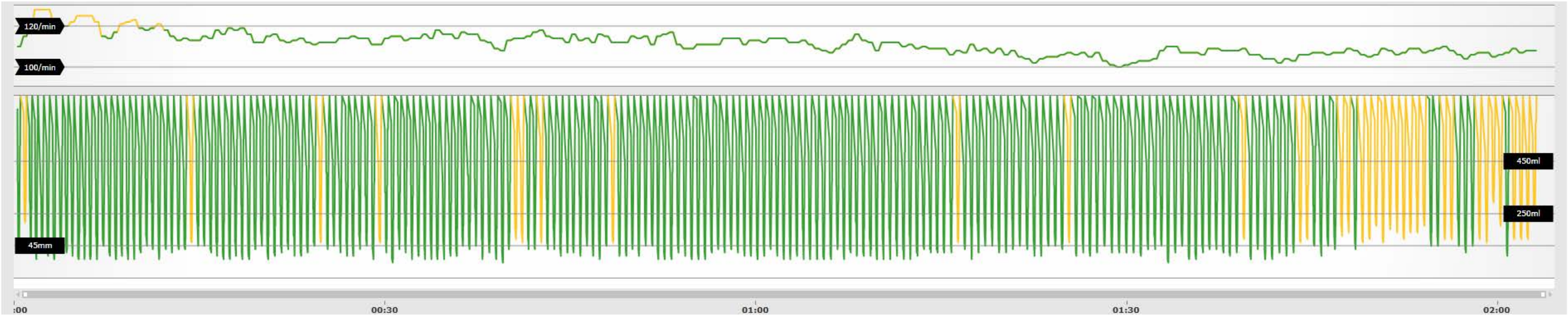

pretest

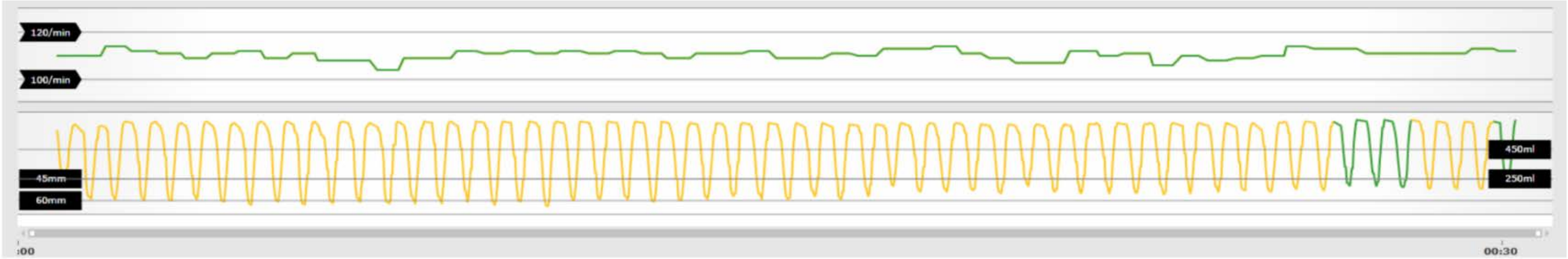

Participant ID: 21, Height 170cm, Weight 55kg, BMI 19kg/cm, age: 40s, sex: female
excluded as a result of pretest

pretest

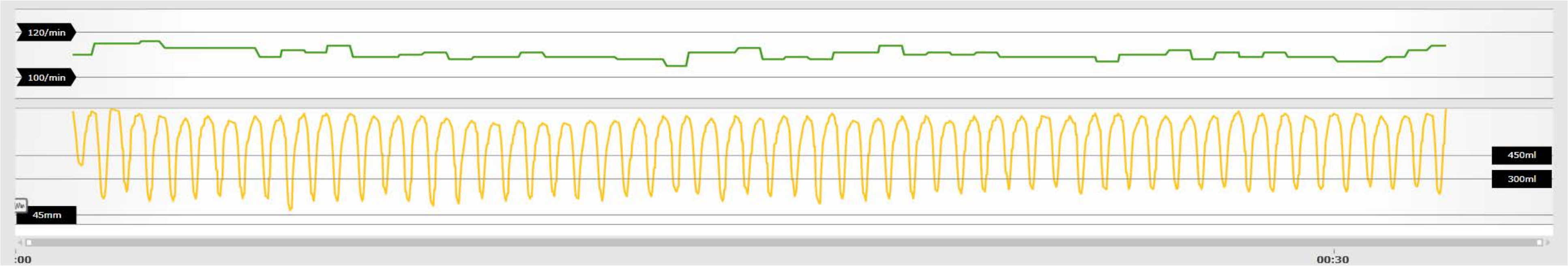

Participant ID: 22, Height 155cm, Weight 45kg, BMI 19kg/cm, age: 20s, sex: female
excluded as a result of pretest

pretest

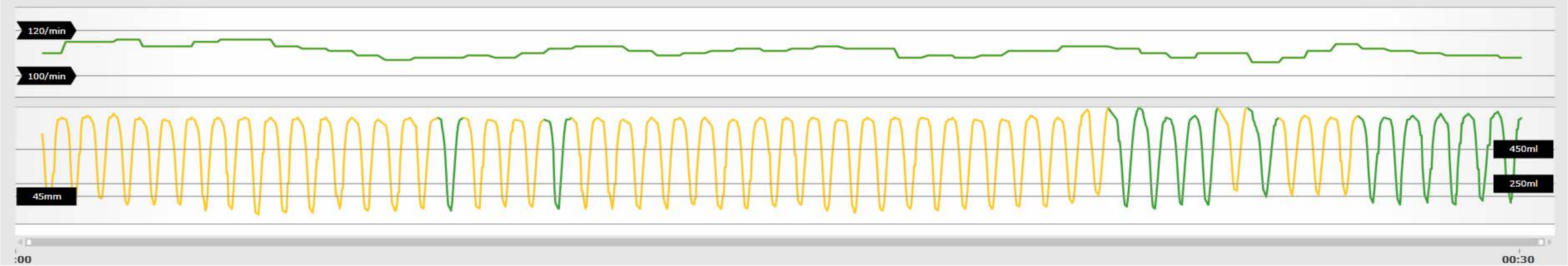

Supplement: S1 Fig — (PDF) [file pone.0216739.s003.pdf]
